# A new extended Fréchet model with different estimation methods and applications

**DOI:** 10.1016/j.heliyon.2024.e36348

**Published:** 2024-08-20

**Authors:** Mohammed Elgarhy, Mohamed Kayid, Ibrahim Elbatal, Mustapha Muhammad

**Affiliations:** a Mathematics and Computer Science Department, Faculty of Science, Beni-Suef University, Beni-Suef 62521, Egypt; b Department of Basic Sciences, Higher Institute of Administrative Sciences, Belbeis, AlSharkia, Egypt; c Department of Statistics and Operations Research, College of Science, King Saud University, P.O. Box 2455, Riyadh 11451, Saudi Arabia; d Faculty of Graduate Studies for Statistical Research, Cairo University, Giza 12613, Egypt; e Department of Mathematics, Guangdong University of Petrochemical Technology, Maoming, China

**Keywords:** Fréchet model, Entropy, Moments, Maximum likelihood estimation, Simulation

## Abstract

In this study, we introduce a new extension of the Fréchet distribution known as the new extended Fréchet (NE_Fr) model. The NE_Fr is created by combining the new extended *X* family of distributions and the Fréchet distribution. The NE_Fr has more flexibility than the classical Fréchet distribution and some generalizations of the Fréchet distribution. The probability density function of the NE_Fr can be decreasing, unimodal and right skewed shape but it's hazard rate function can be decreasing or up-side-down shape. Several mathematical properties of the new model were derived by calculating the quantile function, the ordinary moments, the incomplete moments, the moment generating function, the conditional moment, the Bonferroni curve and the Lorenz curve. Several entropy measures were developed for this purpose. The inferences from the NE_Fr distribution were investigated using several established methods such as maximum likelihood estimation, least squares and weighted least squares estimation, and Anderson-Darling estimation. The simulation results demonstrated the computational efficiency of these techniques. The proposed NE_Fr distribution was demonstrated to be useful by examining three actual data sets.

## 1 Introduction and motivation

### 1.1 Motivation and incitement

Several continuous uni-variate models have been provided recently by statisticians, and they have many applications in fields such as engineering, finance, biological sciences, and economics. Such applications have shown that classically distributed data sets are typically the exception rather than the rule. Therefore, there has been a notable advancement in the generalization of certain well-known models and their effective applications in many useful fields. To improve the flexibility and quality of fits of the distribution against the concept of model parsimony, one or more shape parameter(s) are added to the current probability distribution to generalize the classical distributions.

Maurice Fréchet (1878–1973) introduced the Fréchet (Fr) model [1] and studied it as a potential limit distribution for a series of maxims. The Fr model has another name which is called the inverse Weibull (IW) model. One of the significant distributions in extreme value theory with broad applicability is the Fr distribution (type II extreme value model). It is also known as the inverse Weibull distribution and is a family of continuous distributions developed within the general extreme value theory, which deals with the stochastic behavior of the maximum and the minimum of independent and identically distributed random variables. As specific cases of the Fr distribution, one could consider the inverse Rayleigh and inverse exponential distributions. There are many applications for the Fr distribution, involving stochastic phenomena including rainfall, floods, air pollution, predicting extreme occurrences in hydrology and finance, life testing, earthquakes, and wind speeds, among others. More details about the Fr distribution and its uses may be found in among the following sources such as [2], [3], [4], [5], [6], [7]. The cumulative distribution function (cdf) of the Fr distribution (for z>0) is

(1) $$G(z;\alpha ,\beta )=e^{-\alpha z^{-\beta }}$$

where α>0,β>0 are, respectively, the scale and shape parameters. The corresponding probability density function (pdf) is given by

(2) $$g(z;\alpha ,\beta )=\alpha \beta z^{-\beta -1}e^{-\alpha z^{-\beta }},$$

also, the hazard rate function (hrf) of the Fr distribution given

$$h(z;\alpha ,\beta )=\frac{\alpha \beta z^{-\beta -1}e^{-\alpha z^{-\beta }}}{1-e^{-\alpha z^{-\beta }}}.$$

The pdf (2) exhibits an unimodal shape or right skewed shape depending on whether its hrf is always unimodal.

Over the past few decades, several models have not been flexible enough to provide the best fit for real-world data sets. It is recommended that researchers build an expanded class of probability distributions by adding one or more parameters to standard probability distributions. Due to their popularity in simulating real-life events occurring from a wide range of real-life settings, more focus has been placed on studying the tail behavior of distributions by integrating shape properties. The previously mentioned novel statistical distributions offer increased adaptability when examining diverse applications, including but not limited to life expectancy, biomedical sciences, actuarial sciences, survival analysis, and insurance. Ref. [8] introduced an important family to obtain more flexibility to the baseline distributions with cdf G(z;ξ) which may depended on the vector parameter *ξ*. This family is called a new extended *X* family of distribution. A random variable *Z* is said to follow the proposed family if its cdf is given by

(3) $$F(z;\lambda ,\xi )=1-{\left[\frac{1-G^{2}(z;\xi )}{1-\bar{\lambda }G^{2}(z;\xi )}\right]}^{\lambda },\lambda >0,$$

where the additional parameter *λ* in (3) adds greater distributional flexibility. The pdf corresponding to (3) is

(4) $$f(z;\lambda ,\xi )=\frac{2\lambda^{2}g(z;\xi )G(z;\xi ){\left(1-G^{2}(z;\xi )\right)}^{\lambda -1}}{{\left(1-\bar{\lambda }G^{2}(z;\xi )\right)}^{\lambda +1}}.$$

Also, the hrf is given by

$$\tau (z;\lambda ,\xi )=\frac{2\lambda^{2}g(z;\xi )G(z;\xi )}{\left(1-G^{2}(z;\xi )\right)\left(1-\bar{\lambda }G^{2}(z;\xi )\right)}.$$

Ref. [8] proposed a versatile three-parameter distribution, called a new extended Weibull (NE−W) distribution. The applicability of the proposed distribution has been illustrated via three data sets from medical, engineering, and financial sciences, and the model performs reasonably well as compared to some well-known distributions. In a similar way, in this article, a new extension of the F distribution, called a new extended Fréchet (NE_Fr) distribution to increase the flexibility of the parent model and study basic properties. Some of motivations behind the NE_Fr distribution are to create different types of shapes for pdf and hrf to increase the flexibility for distribution, it can be used in a variety of problems in different areas such as financial, biomedical, survival analysis and industrial reliability.

### 1.2 Literature review

Numerous researchers have suggested different approaches for adding a parameter into probability distributions. These creative families have been utilized to examine modeling data in various practical fields, including engineering, economics, biological research, environmental sciences, and many other disciplines. Some widely recognized families of distributions are the unit exponentiated half logistic power series-G in [9], Dinesh-Umesh-Sanjay (DUS)-G in [10], Kavya-Manoharan Weibull-G in [11], generalized DUS-G in [12], sine-Burr X-G in [13], Cos-G in [14], Type I half logistic Burr X-G in [15], sine Kumaraswamy-G in [16], new extended cosine-G in [17], truncated Cauchy power Weibull-G in [18], Marshall-Olkin odd Burr III-G in [19], Weibull-G in [20], Compounded bell-G in [21], Kavya-Manoharan (KM)-G in [22], Gompertz-G in [23], Type I half-logistic-G in [24], Type II half-logistic-G in [25], a new power Topp-Leone-G in [26], odd Perks-G in [27], odd inverse power generalized Weibull-G in [28], T-X family in [29], sec-G in [30], sine exponentiated Weibull-G in [31], Type II half-logistic odd Fréchet-G in [32], beta-G in [33], generalized odd Burr III-G in [34], odd generalized N-H-G in [35], logarithmically-exponential-G in [36], generalized inverted Kumaraswamy-G in [37], Type II exponentiated half logistic-G in [38], generalized truncated Fréchet-G in [39], anew truncated Muth-G in [40], and for more information see [41], [42], [43], [44], [45].

Several authors investigated some extensions of the Fr or IW model, such as the Marshall-Olkin Fr (MO_Fr) in [46], beta Fr in [47], Kumaraswamy Marshall-Olkin Fr in [48], transmuted exponentiated generalized Fr in [49], Weibull Fr in [50], Kumaraswamy transmuted Marshall-Olkin Fr in [51], transmuted Topp Leone Fr in [52], odd log-logistic Fr in [53], odd Lindley Fr in [54], xgamma Fr in [55], extended IW in [56], new modified IW in [57], Lomax IW in [58], modified Burr XII IW in [59] new half-logistic Fr (NHL_Fr) [60], beta Fr (B_Fr) [61], exponentiated generalized Fr (EG_Fr) [62], half-logistic Fr (HL_Fr) [63], exponential Fr (E_Fr) [64], gamma extended Fr (Ga_Fr) [65], Topp-Leone inverse Weibull (TL_Fr) [66], for more information see [67], [68], [69], [70].

### 1.3 Contribution and paper organization

The reasons behind formulating the NE_Fr distribution are:

• The pdf of the NE_Fr distribution can be unimodal, decreasing, and right-skewed. Furthermore, but the hrf can be decreasing or up-side-down shaped.
• A closed form can be used to express the corresponding quantile and median.
• In fact, several statistical properties such as ordinary moments, incomplete moments, conditional moments, mean, variance, standard deviation, skewness, kurtosis, coefficient of variation, index of dispersion, mean residual lifetime function, mean inactivity time and Bonferroni curves.
• Some measures of uncertainty, like the Rényi entropy, q entropy, Havrda and Charvat entropy and Arimoto entropy can be computed.
• Four existing parametric estimation approaches can be considered to the NE_Fr distribution such as the maximum likelihood (ML), least squares (LS), weighted least squares (WLS) and Anderson-Darling (AD) approaches.
• The NE_Fr distribution outperforms the direct contenders in terms of unit data fit because of its adaptable properties. In this study, its relevance is emphasized by an examination of many existing statistical models, including the new half-logistic Fréchet, beta Fréchet, exponentiated generalized Fréchet, half-logistic Fréchet, exponential Fréchet, gamma extended Fréchet, Topp-Leone inverse Weibull, Marshall Olkin Fréchet, and Fr models and three applications that use real-world data sets.

The remainder of this study is structured as follows. In Section 2, we define the NE_Fr distribution, provide plots for its pdf and hrf, and derive a useful linear representation for its pdf. The mathematical and statistical properties of the proposed distribution are described in Section 3. In Section 4, we introduce four entropy measures. Section 5 presents four different estimation methods: ML, LS, WLS and AD for estimating the unknown parameters for the NE_Fr distribution. In Section 6, a simulation study is conducted to evaluate the behavior of the parameter model using the four estimation approaches. Section 7 presents three real-world applications to investigate the usefulness and flexibility of the model compared to some well-known models. Finally, Section 8 presents concluding remarks.

## 2 The NE_Fr distribution

In this section, we introduce the NE_Fr distribution by taking the cdf of the Fréchet distribution as a baseline in Equation (3). Let a random variable *Z* follows the NE_Fr distribution with three parameters α>0, β>0 and λ>0, if it has the cdf

(5) $$F(z;\lambda ,\alpha ,\beta )=1-{\left[\frac{1-e^{-2\alpha z^{-\beta }}}{1-\bar{\lambda }e^{-2\alpha z^{-\beta }}}\right]}^{\lambda }.$$

The corresponding pdf is constructed by inserting (1) and (2) in (4) as below

(6) $$f(z;\lambda ,\alpha ,\beta )=\frac{2\lambda^{2}\alpha \beta z^{-\beta -1}e^{-2\alpha z^{-\beta }}{\left(1-e^{-2\alpha z^{-\beta }}\right)}^{\lambda -1}}{{\left(1-\bar{\lambda }e^{-2\alpha z^{-\beta }}\right)}^{\lambda +1}}$$

Hereafter, we denote a random variable *Z* with pdf (6) and we write *Z* ∼ NE_Fr(λ,α,β). The survival function and hrf of NE_Fr(λ,α,β) are given respectively by

$$S(z;\lambda ,\alpha ,\beta )={\left[\frac{1-e^{-2\alpha z^{-\beta }}}{1-\bar{\lambda }e^{-2\alpha z^{-\beta }}}\right]}^{\lambda },$$

and

$$\tau (z;\lambda ,\alpha ,\beta )=\frac{2\lambda^{2}\alpha \beta z^{-\beta -1}e^{-2\alpha z^{-\beta }}}{\left(1-e^{-2\alpha z^{-\beta }})\right)\left(1-\bar{\lambda }e^{-2\alpha z^{-\beta }}\right)}.$$

**Figure 1:**
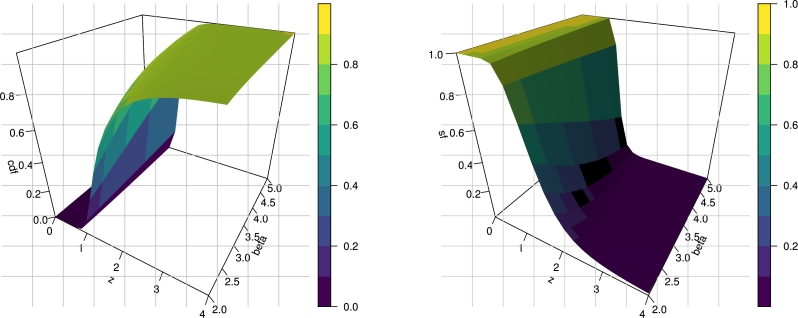
3D Plots of the cdf and sf for the NE_Fr distribution at *α* = *λ* = 2.0.

**Figure 2:**
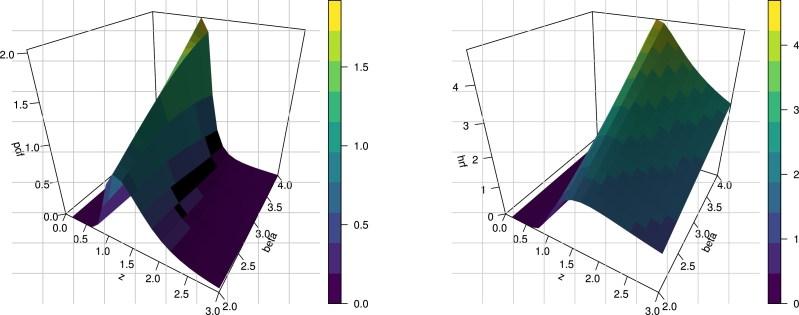
3D Plots of the pdf and hrf for the NE_Fr distribution at *α* = *λ* = 2.0.

**Figure 3:**
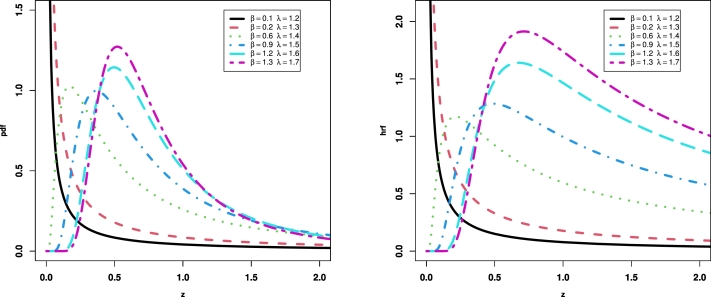
Plots of the pdf and hrf of the NE_Fr distribution at *α* = 0.5.

Figure 1, Figure 2 show 3D plots the cdf, sf, pdf and hrf for the NE_Fr distribution at α=λ=2.0. Fig. 3 shows the pdf and hrf curves for the NE_Fr distribution with different values of *β* and *λ* and at *α* = 0.5. Then we can note that from Fig. 3 the pdf of the NE_Fr model can be decreased, unimodal or right skewed shape. Also, from Fig. 3 the hrf of the NE_Fr model can be decreased or up-side-down shape.

Moreover, we derive an explicit linear representation of the pdf by using the generalized binomial expansions

$${\left(1-x\right)}^{-\delta }=\sum_{i=0}^{\infty }\frac{\Gamma (\delta +i)}{\Gamma (\delta )i!}x^{i},\left|x\right|<1,\delta >0,$$

$${\left(1-x\right)}^{\delta -1}=\sum_{j=0}^{\infty }{\left(-1\right)}^{j}\left(\begin{array}{c} \delta -1\\ j \end{array}\right)x^{j},$$

we can rewrite the pdf (6), as

(7) $$f(z;\lambda ,\alpha ,\beta )=\sum_{i,j=0}^{\infty }\varpi_{i,j}\;z^{-\beta -1}e^{-2((i+j)+1)\alpha z^{-\beta }},$$

where

$$\varpi_{i,j}=2\lambda^{2}\alpha \beta {\left(-1\right)}^{j}\frac{\Gamma (\delta +i)}{\Gamma (\delta )i!}\left(\begin{array}{c} \delta -1\\ j \end{array}\right){\bar{\lambda }}^{i}.$$

## 3 Statistical properties

Quantiles, ordinary moments, the moment-generating function, conditional moments, the Bonferroni curve, and the Lorenz curve are some of the major mathematical and statistical aspects of the NE_Fr distribution that are discussed in this section.

### 3.1 Quantile function

The $p^{th}$ quantile $x_{p}$ of the NE_Fr distribution is given by

$$Q(p)=F^{-1}(p)={\left\{-\frac{1}{2\alpha }\log\left(\frac{1-{\left(1-u\right)}^{\frac{1}{\lambda }}}{1-\bar{\lambda }{\left(1-u\right)}^{\frac{1}{\lambda }}}\right)\right\}}^{-\frac{1}{\beta }},0<p<1.$$

Table 1 show the results of *Q*1, *Q*2, *Q*3, *BSK*, and MKUR associated with the NE_Fr distribution. The values of *Q*1 and *Q*3 increase with higher *α* and *λ*, indicating a broader data distribution, while *Q*2 (the median) consistently rises, showing the central data point moving upwards. *BSK* decreases gradually with increasing *α* and *β*, indicating more concentrated data variance. MKUR decreases with higher *α* and *β*, suggesting the data distribution becomes less skewed and closer to normal.

**Table 1:** Results of *Q*1, *Q*2, *Q*3, *BSK* and *MKUR* associated with the NE_Fr distribution.

*α*	*β*	*λ*	*Q*1	*Q*2	*Q*3	*BSK*	*MKUR*
0.4	0.4	0.9	0.34770	2.29081	25.35068	0.84457	8.10270
		1.3	0.12595	0.51180	2.93203	0.72499	4.40295
		1.7	0.06950	0.21613	0.84938	0.62397	3.09573
		2.1	0.04657	0.12273	0.38057	0.54394	2.47137
		2.3	0.03980	0.09869	0.28065	0.51104	2.27476
						
	0.8	0.9	0.58966	1.51354	5.03495	0.58433	2.80456
		1.3	0.35491	0.71543	1.71232	0.46880	2.10715
		1.7	0.26365	0.46491	0.92161	0.38822	1.80162
		2.1	0.21581	0.35032	0.61691	0.32928	1.63378
		2.3	0.19950	0.31413	0.52978	0.30585	1.57656
						
	1.2	0.9	0.70318	1.31825	2.93763	0.44947	2.02525
		1.3	0.50125	0.79990	1.43127	0.35776	1.70656
		1.7	0.41114	0.60011	0.94703	0.29477	1.55597
		2.1	0.35977	0.49692	0.72469	0.24833	1.46772
		2.3	0.34143	0.46213	0.65473	0.22951	1.43646
						
	1.4	0.9	0.73946	1.26723	2.51849	0.40668	1.85967
		1.3	0.55324	0.82583	1.35981	0.32407	1.61723
		1.7	0.46681	0.64555	0.95443	0.26689	1.49971
		2.1	0.41634	0.54914	0.75880	0.22443	1.42928
		2.3	0.39808	0.51599	0.69557	0.20733	1.40385

0.6	0.4	0.9	0.95815	6.31274	69.85825	0.84457	8.10270
		1.3	0.34709	1.41041	8.07972	0.72498	4.40296
		1.7	0.19153	0.59562	2.34069	0.62396	3.09568
		2.1	0.12834	0.33814	1.04874	0.54412	2.47143
		2.3	0.10969	0.27194	0.77337	0.51104	2.27472
						
	0.8	0.9	0.97886	2.51252	8.35813	0.58433	2.80456
		1.3	0.58913	1.18760	2.84249	0.46881	2.10714
		1.7	0.43763	0.77177	1.52991	0.38818	1.80156
		2.1	0.35823	0.58149	1.02408	0.32938	1.63388
		2.3	0.33119	0.52149	0.87942	0.30577	1.57671
						
	1.2	0.9	0.98582	1.84816	4.11853	0.44946	2.02523
		1.3	0.70277	1.12146	2.00662	0.35776	1.70657
		1.7	0.57642	0.84136	1.32775	0.29475	1.55590
		2.1	0.50439	0.69667	1.01599	0.24831	1.46775
		2.3	0.47868	0.64788	0.91788	0.22948	1.43648
						
	1.4	0.9	0.98789	1.69292	3.36451	0.40669	1.85965
		1.3	0.73909	1.10324	1.81659	0.32408	1.61718
		1.7	0.62362	0.86239	1.27505	0.26691	1.49975
		2.1	0.55618	0.73359	1.01369	0.22447	1.42920
		2.3	0.53181	0.68932	0.92918	0.20725	1.40402

### 3.2 Different types of moments

The $r^{th}$ moment of the NE_Fr distribution is obtained by

$$\mu_{r}^{{}^{\prime }}=E(Z^{r})=\int_{0}^{\infty }z^{r}f(z)dz=\sum_{i,j=0}^{\infty }\varpi_{i,j}\int_{0}^{\infty }z^{-\beta -1}e^{-2((i+j)+1)\alpha z^{-\beta }}dz,$$

setting y=2((i+j)+1)α $z^{-\beta }$ and after some algebra we get

$$\mu_{r}^{{}^{\prime }}=\sum_{i,j=0}^{\infty }\varpi_{i,j}\frac{\Gamma (1-\frac{r}{\beta })}{\beta {\left[2(i+j+1)\right]}^{1-\frac{r}{\beta }}}.$$

The moment generating function is given by $M_{Z}(t)=\int_{0}^{\infty }e^{-tz}f(z)dz$, using the Taylors series expansion of the function $e^{-tz}$, we get

$$M_{Z}(t)=\int_{0}^{\infty }e^{-tz}f(z)dz=\int_{0}^{\infty }\sum_{k=0}^{\infty }\frac{t^{k}}{k!}\mu_{k}^{{}^{\prime }}=\sum_{k=0}^{\infty }\sum_{i,j=0}^{\infty }\frac{t^{k}}{k!}\varpi_{i,j}\frac{\Gamma (1-\frac{r}{\beta })}{\beta {\left[2(i+j+1)\right]}^{1-\frac{r}{\beta }}}.$$

The incomplete moments for NE_Fr distribution is obtained by

$$\psi_{s}(t)=E(Z^{{}^{s}}|Z<t)=\int_{0}^{t}z^{s}f(z)dz=\sum_{i,j=0}^{\infty }\varpi_{i,j}\int_{0}^{t}z^{-\beta -1}e^{-2((i+j)+1)\alpha z^{-\beta }}dz=\sum_{i,j=0}^{\infty }\varpi_{i,j}\frac{\gamma (\frac{s}{\beta }+1,2(i+j+1)\alpha t^{-\beta })}{\beta {\left[2(i+j+1)\right]}^{1-\frac{s}{\beta }}},$$

where $\gamma (s,t)=\int_{0}^{t}x^{s-1}e^{-x}dx$ is the lower incomplete gamma function. Some numerical results of the mean ($\mu_{1}^{\prime }$), variance ($\sigma^{2}$), standard deviation (*σ*), skewness (S), kurtosis (K), coefficient of variation (CV) and index of dispersion (ID) are provided in Table 2.

**Table 2:** Results of $\mu_{1}^{\prime }$, *σ*^2^, *σ*, S, K, CV and ID for the NE_Fr distribution.

*α*	*β*	*λ*	$\mu_{1}^{\prime }$	*σ*^2^	*σ*	*S*	*K*	*CV*	*ID*
0.6	0.2	0.5	7.08740	263.67959	16.23821	3.28156	14.19987	2.29114	0.43646
		0.9	6.38569	193.71802	13.91826	3.73932	18.52443	2.17960	0.45880
		1.3	4.74989	113.52809	10.65496	4.81869	30.63455	2.24320	0.44579
		1.7	3.48394	60.94547	7.80676	6.30259	53.36341	2.24079	0.44627
		2.1	2.66790	31.60306	5.62166	8.10945	92.20319	2.10715	0.47457
								
	0.4	0.5	9.35976	344.96140	18.57314	2.70838	10.19348	1.98436	0.50394
		0.9	8.94200	266.34491	16.32008	3.00422	12.59383	1.82510	0.54792
		1.3	6.65506	159.73988	12.63882	3.92090	20.87975	1.89913	0.52656
		1.7	4.78914	86.86192	9.31997	5.19923	36.76051	1.94606	0.51386
		2.1	3.56819	45.28950	6.72975	6.77038	64.30638	1.88604	0.53021
								
	0.8	0.5	11.24012	423.61748	20.58197	2.32701	7.91563	1.83112	0.54611
		0.9	12.08208	354.11612	18.81797	2.40199	8.70315	1.55751	0.64205
		1.3	9.16514	219.48719	14.81510	3.17654	14.33379	1.61646	0.61864
		1.7	6.52017	121.73943	11.03356	4.28584	25.48904	1.69222	0.59094
		2.1	4.74682	64.12066	8.00754	5.65924	45.14608	1.68693	0.59279
								
	1.2	0.5	11.73149	459.61510	21.43864	2.21182	7.25333	1.82744	0.54721
		0.9	14.07944	411.43511	20.28386	2.10274	7.09243	1.44067	0.69412
		1.3	10.94090	260.97835	16.15482	2.79846	11.54566	1.47655	0.67725
		1.7	7.76864	146.96233	12.12280	3.82349	20.63669	1.56048	0.64083
		2.1	5.59321	78.06997	8.83572	5.09674	36.81862	1.57972	0.63302

1.3	0.2	0.5	1.64765	47.37715	6.88311	8.16362	82.71369	4.17753	0.23938
		0.9	1.02588	12.45366	3.52897	13.35883	245.67998	3.43995	0.29070
		1.3	0.82996	3.15624	1.77658	19.11596	623.41644	2.14057	0.46717
		1.7	0.78776	0.98983	0.99490	19.89656	967.07445	1.26295	0.79180
		2.1	0.78824	0.42152	0.64925	14.77203	820.88782	0.82366	1.21409
								
	0.4	0.5	3.60575	105.38020	10.26549	5.29206	35.68592	2.84697	0.35125
		0.9	2.01182	28.46234	5.33501	8.89603	108.37296	2.65183	0.37710
		1.3	1.39785	7.04437	2.65412	13.43700	295.97519	1.89872	0.52667
		1.7	1.18289	2.06602	1.43737	15.39089	528.93396	1.21513	0.82296
		2.1	1.09637	0.80117	0.89508	12.81809	540.91314	0.81641	1.22487
								
	0.8	0.5	7.33678	214.03280	14.62986	3.44538	16.10648	1.99404	0.50149
		0.9	3.84587	62.18856	7.88597	5.98835	49.36632	2.05051	0.48768
		1.3	2.34134	15.31599	3.91356	9.54978	144.20943	1.67151	0.59826
		1.7	1.77482	4.25232	2.06212	11.93829	292.96541	1.16187	0.86068
		2.1	1.52481	1.51490	1.23081	11.06342	356.15131	0.80719	1.23887
								
	1.2	0.5	10.54379	304.72080	17.45625	2.68031	10.44202	1.65560	0.60401
		0.9	5.52997	95.36388	9.76544	4.76631	31.69539	1.76591	0.56628
		1.3	3.15456	23.75371	4.87378	7.85497	95.90023	1.54499	0.64725
		1.7	2.24902	6.43546	2.53682	10.29931	208.60175	1.12797	0.88655
		2.1	1.84921	2.19235	1.48066	10.12312	278.76734	0.80070	1.24891

Table 2 shows how statistical values change with varying parameters *α*, *β*, and *λ*. Both $\mu_{1}^{\prime }$ and *σ* increase with higher *α* and *λ*. *S* decreases with higher *α* and *β*, while *K* increases with higher *β* and *λ*.

### 3.3 Conditional moments

It is also of relevance to discover the conditional moments and the mean residual lifetime function when dealing with lifespan models. To determine the conditional moments for the NE_Fr distribution, the following is done:

$$E(Z^{{}^{s}}|Z>t)=\frac{1}{\bar{F}(t)}\zeta_{s}(t)$$

$$\zeta_{s}(t)=\int_{t}^{\infty }z^{s}f(z)dz=\sum_{i,j=0}^{\infty }\varpi_{i,j}\int_{t}^{\infty }z^{-\beta -1}e^{-2((i+j)+1)\alpha z^{-\beta }}dz=\sum_{i,j=0}^{\infty }\varpi_{i,j}\frac{\Gamma (\frac{s}{\beta }+1,2(i+j+1)\alpha t^{-\beta })}{\beta {\left[2(i+j+1)\right]}^{1-\frac{s}{\beta }}},$$

where $\Gamma (s,t)=\int_{t}^{\infty }x^{s-1}e^{-x}dx$ is the upper incomplete gamma function. The average remaining lifespan for a person who arrived at age *t* is referred to as the mean residual lifetime function (MRL), which is also referred to as the life expectancy at age *t* or the life expectancy, respectively. Applications include the fields of economics, maintenance, product quality control, and maintenance, as well as the fields of life insurance and medical sciences. This is provided by

$$\mu (t)=\frac{1}{\bar{F}(t)}\int_{t}^{\infty }zf(z)dz-t=\frac{1}{\bar{F}(t)}\sum_{i,j=0}^{\infty }\varpi_{i,j}\frac{\Gamma (\frac{1}{\beta }+1,2(i+j+1)\alpha t^{-\beta })}{\beta {\left[2(i+j+1)\right]}^{1-\frac{1}{\beta }}}-t.$$

Additionally, the inverted mean residual lifetime, also known as the mean inactivity time (MIT), is the amount of time that has passed after the failure of an item, assuming that this failure occurred between the time intervals of (0;t). The MIT is provided by

$$M(t)=t-\frac{1}{F(t)}\int_{0}^{t}zf(z)dz=t-\frac{1}{F(t)}\sum_{i,j=0}^{\infty }\varpi_{i,j}\frac{\gamma (\frac{1}{\beta }+1,2(i+j+1)\alpha t^{-\beta })}{\beta {\left[2(i+j+1)\right]}^{1-\frac{1}{\beta }}}.$$

### 3.4 Bonferroni and Lorenz curves

The Bonferroni and Lorenz Curves were suggested while we were working on this part. The Bonferroni and Lorenz curves [71] and the Bonferroni and Gini indices have applications not just in the area of economics, where they are used to examine income and poverty, but also in other domains such as reliability, demography, insurance, and medicine. Two curves define the Bonferroni and Lorenz curves:

$$B(p)=\frac{1}{p\mu }\int_{0}^{q}zf(z)dz=\frac{1}{p\mu }\sum_{i,j=0}^{\infty }\varpi_{i,j}\frac{\gamma (\frac{1}{\beta }+1,2(i+j+1)\alpha q^{-\beta })}{\beta {\left[2(i+j+1)\right]}^{1-\frac{1}{\beta }}},$$

and

$$L(p)=\frac{1}{\mu }\int_{0}^{q}zf(z)dz=\frac{1}{\mu }\sum_{i,j=0}^{\infty }\varpi_{i,j}\frac{\gamma (\frac{1}{\beta }+1,2(i+j+1)\alpha q^{-\beta })}{\beta {\left[2(i+j+1)\right]}^{1-\frac{1}{\beta }}}.$$

### 3.5 Order statistics

Assume $z_{1},\;z_{2},\;\ldots ,z_{n}$ be random sample from size *n* and follows the NE_Fr distribution with cdf (5) and pdf (6). Suppose that $z_{\left(1\right)},\;z_{\left(2\right)},\;\ldots ,z_{\left(n\right)}$ be the observed order statistics. The pdf of *m*th order statistics is provided via:

(8) $$f_{Z_{\left(m\right)}}(z)=\frac{n!}{(m-1)!(n-m)!}f(z){\left[F(z)\right]}^{m-1}{\left[1-F(z)\right]}^{n-m}.$$

By employing (6) and (5) in (8), we have the pdf of $Z_{\left(m\right)}$ of order statistics for the NE_Fr distribution as below:

(9) $$f_{Z_{\left(m\right)}}(z)=\frac{2n!\lambda^{2}\alpha \beta }{(m-1)!(n-m)!}\times \frac{z^{-\beta -1}e^{-2\alpha z^{-\beta }}{\left(1-e^{-2\alpha z^{-\beta }}\right)}^{\lambda (n-m+1)-1}}{{\left(1-\bar{\lambda }e^{-2\alpha z^{-\beta }}\right)}^{\lambda (n-m+1)+1}}\times {\left[1-{\left[\frac{1-e^{-2\alpha z^{-\beta }}}{1-\bar{\lambda }e^{-2\alpha z^{-\beta }}}\right]}^{\lambda }\right]}^{m-1}.$$

By putting *m* = 1 and m=n in (9), we have the lowest order statistics and the last order statistics for the NE_Fr distribution as below:

$$f_{Z_{\left(1\right)}}(z)=\frac{2n\lambda^{2}\alpha \beta z^{-\beta -1}e^{-2\alpha z^{-\beta }}{\left(1-e^{-2\alpha z^{-\beta }}\right)}^{\lambda n-1}}{{\left(1-\bar{\lambda }e^{-2\alpha z^{-\beta }}\right)}^{\lambda n+1}},$$

and

$$f_{Z_{\left(n\right)}}(z)=\frac{2n\lambda^{2}\alpha \beta z^{-\beta -1}e^{-2\alpha z^{-\beta }}{\left(1-e^{-2\alpha z^{-\beta }}\right)}^{\lambda -1}}{{\left(1-\bar{\lambda }e^{-2\alpha z^{-\beta }}\right)}^{\lambda +1}}\times {\left[1-{\left[\frac{1-e^{-2\alpha z^{-\beta }}}{1-\bar{\lambda }e^{-2\alpha z^{-\beta }}}\right]}^{\lambda }\right]}^{n-1}.$$

## 4 Entropy

Entropy is used to measure the randomness of systems, and it is widely used in fields such as physics, molecular imaging of tumors, and sparse kernel density estimation. If *Z* has the pdf, Rényi entropy (RE) [73] can be defined as

$$I_{R}(\rho )=\frac{1}{1-\rho }\log\left[I_{\rho }\right],\;\;\;\;\;\rho \neq 1,\rho >0,$$

where

$$I_{\rho }=\int_{0}^{\infty }f^{{}^{{}^{\rho }}}(z)dz=\int_{0}^{\infty }\frac{{\left(2\lambda^{2}\alpha \beta \right)}^{\rho }z^{-\rho (\beta +1)}e^{-2\rho \alpha z^{-\beta }}{\left(1-e^{-2\alpha z^{-\beta }}\right)}^{\rho (\lambda -1)}}{{\left(1-\bar{\lambda }e^{-2\alpha z^{-\beta }}\right)}^{\rho (\lambda +1)}}dz.$$

After some simplifications, we get

$$I_{\rho }=\sum_{i,j=0}^{\infty }\Xi_{i,j}\frac{\Gamma (\rho +\frac{\rho -1}{\beta })}{\beta {\left(2\alpha (\rho +i+j)\right)}^{\rho +\frac{\rho -1}{\beta }}},$$

where $\Xi_{i,j}={\left(2\lambda^{2}\alpha \beta \right)}^{\rho }{\left(-1\right)}^{j}{\bar{\lambda }}^{i}\left(\begin{array}{c} \rho (\lambda +1)+i-1\\ i \end{array}\right)\left(\begin{array}{c} \rho (\lambda -1)\\ j \end{array}\right)$. Thus we can write the RE for the NE_Fr distribution is given by

$$I_{R}(\rho )=\frac{1}{1-\rho }\log\left[\sum_{i,j=0}^{\infty }\Xi_{i,j}\frac{\Gamma (\rho +\frac{\rho -1}{\beta })}{\beta {\left(2\alpha (\rho +i+j)\right)}^{\rho +\frac{\rho -1}{\beta }}}\right].$$

Also, the q-entropy (QE) [74] is defined as

$$T_{\rho }=\frac{1}{\rho -1}\left[1-I_{\rho }\right],\;\rho >0,\rho \neq 1.$$

Then, the QE for the NE_Fr distribution is given by

$$T_{\rho }=\frac{1}{\rho -1}\left[1-\sum_{i,j=0}^{\infty }\Xi_{i,j}\frac{\Gamma (\rho +\frac{\rho -1}{\beta })}{\beta {\left(2\alpha (\rho +i+j)\right)}^{\rho +\frac{\rho -1}{\beta }}}\right],\;\rho >0,\rho \neq 1.$$

The Havrda and Charvat entropy (HaCE) [75] is defined as

$$HC_{\rho }=\frac{1}{2^{1-\rho }-1}\left[{\left(E_{\rho }\left(\lambda ,\delta \right)\right)}^{\frac{1}{\rho }}-1\right],\;\rho >0,\rho \neq 1.$$

Then, the HaCE for the NE_Fr distribution is given by

$$HC_{\rho }=\frac{1}{2^{1-\rho }-1}\left[{\left(\sum_{i,j=0}^{\infty }\Xi_{i,j}\frac{\Gamma (\rho +\frac{\rho -1}{\beta })}{\beta {\left(2\alpha (\rho +i+j)\right)}^{\rho +\frac{\rho -1}{\beta }}}\right)}^{\frac{1}{\rho }}-1\right],\;\rho >0,\rho \neq 1.$$

The Arimoto entropy (ArE) [72] is defined as

$$A_{\rho }=\frac{\rho }{1-\rho }\left[{\left(E_{\rho }\left(\lambda ,\delta \right)\right)}^{\frac{1}{\rho }}-1\right],\;\rho >0,\rho \neq 1.$$

Then, the ArE for the NE_Fr distribution is given by

$$A_{\rho }=\frac{\rho }{1-\rho }\left[{\left(\sum_{i,j=0}^{\infty }\Xi_{i,j}\frac{\Gamma (\rho +\frac{\rho -1}{\beta })}{\beta {\left(2\alpha (\rho +i+j)\right)}^{\rho +\frac{\rho -1}{\beta }}}\right)}^{\frac{1}{\rho }}-1\right],\;\rho >0,\rho \neq 1.$$

Some numerical values of entropy measures of the NE_Fr distribution are reported in Table 3, Table 4. Generally, with increasing *α* and *β*, we observe a decrease in entropy values.

**Table 3:** Numerical values of entropy measures of the NE_Fr distribution.

*α*	*β*	*λ*	ε=0.5				ε=0.8			
			**RE**	**HaCE**	**ArE**	**QE**	**RE**	**HaCE**	**ArE**	**QE**
0.6	0.2	0.5	-1.13815	-1.76303	-0.92725	-1.46054	-4.31464	-5.80295	-3.66627	-4.31444
		0.9	-0.72190	-1.36268	-0.81029	-1.12888	-2.59804	-4.69226	-3.10350	-3.48866
		1.3	-0.51734	-1.08343	-0.69615	-0.89754	-1.71056	-3.66601	-2.50576	-2.72565
		1.7	-0.41613	-0.91898	-0.61641	-0.76131	-1.21574	-2.88313	-2.01333	-2.14358
		2.1	-0.37825	-0.85233	-0.58145	-0.70609	-0.95119	-2.38536	-1.68654	-1.77350
									
	0.4	0.5	-1.04589	-1.69006	-0.91003	-1.40009	-4.14850	-5.72963	-3.63277	-4.25993
		0.9	-0.61420	-1.22387	-0.75689	-1.01388	-2.40420	-4.50246	-2.99767	-3.34754
		1.3	-0.39034	-0.87392	-0.59294	-0.72398	-1.48128	-3.32534	-2.29494	-2.47237
		1.7	-0.26616	-0.63717	-0.45819	-0.52785	-0.94453	-2.37202	-1.67765	-1.76358
		2.1	-0.20174	-0.50037	-0.37156	-0.41452	-0.63188	-1.69793	-1.21971	-1.26239
									
	0.8	0.5	-1.08513	-1.72205	-0.91780	-1.42659	-4.18888	-5.74797	-3.64121	-4.27357
		0.9	-0.64328	-1.26305	-0.77264	-1.04634	-2.42923	-4.52794	-3.01201	-3.36648
		1.3	-0.40680	-0.90282	-0.60807	-0.74792	-1.48722	-3.33464	-2.30076	-2.47927
		1.7	-0.26774	-0.64040	-0.46016	-0.53053	-0.92800	-2.33878	-1.65545	-1.73886
		2.1	-0.18637	-0.46619	-0.34892	-0.38621	-0.58994	-1.59988	-1.15177	-1.18950
									
	1.2	0.5	-1.16085	-1.77984	-0.93095	-1.47446	-4.27360	-5.78536	-3.65829	-4.30137
		0.9	-0.71525	-1.35459	-0.80736	-1.12218	-2.50867	-4.60686	-3.05617	-3.42516
		1.3	-0.47413	-1.01556	-0.66436	-0.84132	-1.56009	-3.44653	-2.37057	-2.56246
		1.7	-0.32965	-0.76244	-0.53189	-0.63162	-0.99319	-2.46850	-1.74180	-1.83531
		2.1	-0.24216	-0.58739	-0.42741	-0.48661	-0.64649	-1.73163	-1.24300	-1.28745

1.3	0.2	0.5	-1.73125	-2.08525	-0.98143	-1.72748	-6.87597	-6.44156	-3.92360	-4.78925
		0.9	-1.23873	-1.83423	-0.94229	-1.51953	-4.90095	-6.02114	-3.76187	-4.47667
		1.3	-0.94282	-1.59882	-0.88593	-1.32451	-3.71057	-5.50721	-3.52748	-4.09457
		1.7	-0.73850	-1.38258	-0.81740	-1.14537	-2.88448	-4.94348	-3.23978	-3.67544
		2.1	-0.58858	-1.18822	-0.74212	-0.98435	-2.27361	-4.36470	-2.91942	-3.24512
									
	0.4	0.5	-1.73331	-2.08603	-0.98152	-1.72812	-6.87046	-6.44084	-3.92336	-4.78871
		0.9	-1.23814	-1.83384	-0.94221	-1.51921	-4.89131	-6.01800	-3.76054	-4.47434
		1.3	-0.93878	-1.59502	-0.88486	-1.32136	-3.69549	-5.49873	-3.52336	-4.08826
		1.7	-0.73020	-1.37267	-0.81388	-1.13716	-2.86269	-4.92551	-3.23018	-3.66207
		2.1	-0.57522	-1.16922	-0.73406	-0.96861	-2.24385	-4.33213	-2.90075	-3.22090
									
	0.8	0.5	-1.84960	-2.12715	-0.98586	-1.76219	-7.00599	-6.45803	-3.92911	-4.80149
		0.9	-1.35287	-1.90565	-0.95563	-1.57869	-5.02464	-6.06011	-3.77823	-4.50564
		1.3	-1.05144	-1.69467	-0.91117	-1.40391	-3.82592	-5.57022	-3.55784	-4.14141
		1.7	-0.84033	-1.49670	-0.85557	-1.23991	-2.98956	-5.02763	-3.28440	-3.73800
		2.1	-0.68236	-1.31369	-0.79220	-1.08830	-2.36651	-4.46355	-2.97569	-3.31861
									
	1.2	0.5	-1.95751	-2.16069	-0.98897	-1.78997	-7.12494	-6.47227	-3.93381	-4.81208
		0.9	-1.46022	-1.96478	-0.96534	-1.62768	-5.14284	-6.09533	-3.79282	-4.53183
		1.3	-1.15806	-1.77779	-0.93051	-1.47277	-3.94314	-5.63090	-3.58669	-4.18653
		1.7	-0.94604	-1.60184	-0.88677	-1.32701	-3.10556	-5.11593	-3.33062	-3.80365
		2.1	-0.78701	-1.43861	-0.83670	-1.19179	-2.48106	-4.57976	-3.04105	-3.40501

**Table 4:** Numerical values of entropy measures of the NE_Fr distribution.

*α*	*β*	*λ*	ε=1.5				ε=2.1			
			**RE**	**HaCE**	**ArE**	**QE**	**RE**	**HaCE**	**ArE**	**QE**
0.6	0.2	0.5	2.67882	3.25794	2.61613	1.90846	1.36389	1.81523	1.54061	0.88036
		0.9	1.28431	2.63594	1.88052	1.54410	0.43449	1.25083	0.77868	0.60663
		1.3	0.48049	1.45066	0.92529	0.84978	-0.12420	-0.69294	-0.30850	-0.33607
		1.7	-0.05726	-0.23266	-0.13479	-0.13629	-0.51575	-5.04717	-1.64709	-2.44780
		2.1	-0.44712	-2.29862	-1.22826	-1.34650	-0.81253	-12.80332	-3.17762	-6.20942
									
	0.4	0.5	3.05593	3.31298	2.71261	1.94070	1.91653	1.85986	1.71989	0.90200
		0.9	1.71726	2.94143	2.19703	1.72305	1.04938	1.74308	1.37063	0.84537
		1.3	0.97887	2.30796	1.58476	1.35197	0.55789	1.41822	0.93500	0.68782
		1.7	0.51205	1.52071	0.97494	0.89081	0.23344	0.83672	0.46848	0.40580
		2.1	0.19461	0.68531	0.41624	0.40145	0.00002	0.00008	0.00004	0.00004
									
	0.8	0.5	3.02148	3.30888	2.70490	1.93830	1.89051	1.85887	1.71385	0.90152
		0.9	1.70883	2.93682	2.19182	1.72035	1.05701	1.74560	1.37557	0.84659
		1.3	1.00239	2.33752	1.61008	1.36928	0.60564	1.47020	0.98952	0.71303
		1.7	0.57195	1.64688	1.06593	0.96472	0.32479	1.05106	0.61876	0.50975
		2.1	0.29344	0.97880	0.60498	0.57337	0.13515	0.54336	0.28715	0.26352
									
	1.2	0.5	2.92524	3.29654	2.68228	1.93107	1.78854	1.85427	1.68830	0.89929
		0.9	1.62076	2.88588	2.13530	1.69051	0.96521	1.71186	1.31310	0.83023
		1.3	0.92452	2.23653	1.52447	1.31013	0.52658	1.38056	0.89751	0.66955
		1.7	0.50594	1.50735	0.96543	0.88299	0.26035	0.90509	0.51448	0.43896
		2.1	0.24044	0.82559	0.50556	0.48362	0.08621	0.36771	0.18854	0.17834

1.3	0.2	0.5	5.06623	3.40421	2.93857	1.99414	3.17275	1.87387	1.86751	0.90880
		0.9	3.57590	3.35858	2.80718	1.96741	2.21966	1.86769	1.77783	0.90580
		1.3	2.67086	3.25650	2.61378	1.90761	1.63740	1.84484	1.64416	0.89472
		1.7	2.03541	3.08643	2.37100	1.80799	1.22501	1.79026	1.47342	0.86825
		2.1	1.55737	2.84588	2.09219	1.66708	0.91104	1.68795	1.27286	0.81863
									
	0.4	0.5	5.09567	3.40454	2.93994	1.99434	3.22562	1.87394	1.87008	0.90883
		0.9	3.61295	3.36090	2.81259	1.96877	2.28263	1.86869	1.78743	0.90629
		1.3	2.71773	3.26479	2.62743	1.91247	1.71341	1.85003	1.66736	0.89724
		1.7	2.09436	3.10794	2.39882	1.82059	1.31691	1.80775	1.51913	0.87673
		2.1	1.63057	2.89181	2.14178	1.69398	1.02146	1.73346	1.35219	0.84070
									
	0.8	0.5	4.93470	3.40257	2.93204	1.99318	3.05162	1.87365	1.86097	0.90869
		0.9	3.45531	3.35029	2.78848	1.96256	2.11269	1.86558	1.75975	0.90478
		1.3	2.56448	3.23595	2.58092	1.89558	1.54886	1.83739	1.61430	0.89111
		1.7	1.94654	3.05112	2.32660	1.78730	1.15907	1.77495	1.43736	0.86083
		2.1	1.48923	2.79949	2.04344	1.63991	0.87165	1.66838	1.24190	0.80914
									
	1.2	0.5	4.80212	3.40065	2.92477	1.99206	2.91246	1.87330	1.85217	0.90852
		0.9	3.32378	3.33984	2.76601	1.95643	1.97473	1.86186	1.73271	0.90298
		1.3	2.43432	3.20714	2.53689	1.87870	1.41249	1.82210	1.56160	0.88369
		1.7	1.81810	2.99325	2.25683	1.75341	1.02470	1.73461	1.35436	0.84126
		2.1	1.36284	2.70321	1.94600	1.58350	0.73970	1.58659	1.12681	0.76947

## 5 Statistical inference

In this section, we will study some different methods to estimate the parameters *α*, *β*, and *λ*, these methods are maximum likelihood estimation (MLE), least squares estimation (LE), weighted least squares (WLSE) estimation and Anderson-Darling (ADE) estimation. Also, the statistical software R will be used to solve the equations resulting from these estimation methods.

### 5.1 Maximum likelihood estimation

Using the MLE is popular in estimating the unknown parameters, it depends on maximizing the logarithm of the likelihood of the distribution, if $z_{1},...,z_{n}$ is a random sample has size *n* of the NE_Fr distribution the log-likelihood function is,

(10) $$L_{n}=n\log(2)+2n\log(\lambda )+n\log(\alpha )+n\log(\beta )-(\beta +1)\sum_{i=1}^{n}z_{i}-2\alpha \sum_{i=1}^{n}z_{i}^{-\beta }+(\lambda -1)\sum_{i=1}^{n}\log(1-e^{-2\alpha z_{i}^{-\beta }})-(\lambda +1)\sum_{i=1}^{n}\log(1-\bar{\lambda }e^{-2\alpha z_{i}^{-\beta }}).$$

The MLE uses the partial derivative of $L_{n}$ to its parameters *α*, *β*, and *λ*, and equates these equations by zero then find the solutions using the suitable software which is R in our paper, the partial derivatives of equation (10) are:

$$\frac{\partial L_{n}}{\partial \lambda }=\frac{2n}{\lambda }+\sum_{i=1}^{n}\log(1-e^{-2\alpha z_{i}^{-\beta }})-\sum_{i=1}^{n}log(1-\bar{\lambda }e^{-2\alpha z_{i}^{-\beta }})-\frac{\left(\lambda +1)e^{-2\alpha z_{i}^{-\beta }}\right)}{1-\bar{\lambda }e^{-2\alpha z_{i}^{-\beta }}},$$

$$\frac{\partial L_{n}}{\partial \beta }=\frac{n}{\beta }-\sum_{i=1}^{n}\log(z_{i})-2\alpha \sum_{i=1}^{n}z_{i}^{-\beta }\log(z_{i})+\sum_{i=1}^{n}\frac{2(\lambda -1)\alpha z_{i}^{-\beta }\log(z_{i})e^{-2\alpha z_{i}^{-\beta }}}{1-e^{-2\alpha z_{i}^{-\beta }}}-\sum_{i=1}^{n}\frac{2(\lambda +1)\bar{\lambda }\alpha z_{i}^{-\beta }\log(z_{i})e^{-2\alpha z_{i}^{-\beta }}}{1-\bar{\lambda }e^{-2\alpha z_{i}^{-\beta }}},$$

and

$$\frac{\partial L_{n}}{\partial \alpha }=\frac{n}{\alpha }-2\sum_{i=1}^{n}z_{i}^{-\beta }+\sum_{i=1}^{n}\frac{2(\lambda -1)z_{i}^{-\beta }e^{-2\alpha z_{i}^{-\beta }}}{1-e^{-2\alpha z_{i}^{-\beta }}}-\sum_{i=1}^{n}\frac{2(\lambda +1)\bar{\lambda }z_{i}^{-\beta }e^{-2\alpha z_{i}^{-\beta }}}{1-\bar{\lambda }e^{-2\alpha z_{i}^{-\beta }}}.$$

### 5.2 Least squares method

This method of least squares was introduced by [76], it minimizes its functions to get the estimates of the parameters, and the least squares function is defined as:

(11) $$LSE(\lambda ,\alpha ,\beta )=\sum_{i=1}^{n}{\left[F(z_{\left(i\right)},\alpha ,\beta ,\lambda )-\frac{i}{n+1}\right]}^{2}=\sum_{i=1}^{n}{\left[1-{\left[\frac{1-e^{-2\alpha z_{\left(i\right)}^{-\beta }}}{1-\bar{\lambda }e^{-2\alpha z_{\left(i\right)}^{-\beta }}}\right]}^{\lambda }-\frac{i}{n+1}\right]}^{2}.$$

The partial derivatives of (11) to the parameters *λ*, *α* and *β* are:

$$\frac{\partial LSE(\lambda ,\alpha ,\beta )}{\partial \lambda }=2\lambda \sum_{i=1}^{n}\left[1-{\left[\frac{1-e^{-2\alpha z_{i}^{-\beta }}}{1-\bar{\lambda }e^{-2\alpha z_{i}^{-\beta }}}\right]}^{\lambda }-\frac{i}{n+1}\right]\frac{e^{-2\alpha z_{i}^{-\beta }}{\left[1-e^{-2\alpha z_{i}^{-\beta }}\right]}^{\lambda }}{{\left[1-\bar{\lambda }e^{-2\alpha z_{i}^{-\beta }}\right]}^{\lambda +1}},$$

$$\frac{\partial LSE(\lambda ,\alpha ,\beta )}{\partial \alpha }=4\lambda^{2}\sum_{i=1}^{n}\left[1-{\left[\frac{1-e^{-2\alpha z_{i}^{-\beta }}}{1-\bar{\lambda }e^{-2\alpha z_{i}^{-\beta }}}\right]}^{\lambda }-\frac{i}{n+1}\right]\left[-{\left[\frac{1-e^{-2\alpha z_{i}^{-\beta }}}{1-\bar{\lambda }e^{-2\alpha z_{i}^{-\beta }}}\right]}^{\lambda -1}\left[\frac{z_{i}^{-\beta }e^{-2\alpha z_{i}^{-\beta }}}{{\left[1-\bar{\lambda }e^{-2\alpha z_{i}^{-\beta }}\right]}^{2}}\right]\right],$$

and

$$\frac{\partial LSE(\lambda ,\alpha ,\beta )}{\partial \beta }=4\alpha \lambda^{2}\sum_{i=1}^{n}\left[1-{\left[\frac{1-e^{-2\alpha z_{i}^{-\beta }}}{1-\bar{\lambda }e^{-2\alpha z_{i}^{-\beta }}}\right]}^{\lambda }-\frac{i}{n+1}\right]\left[-{\left[\frac{1-e^{-2\alpha z_{i}^{-\beta }}}{1-\bar{\lambda }e^{-2\alpha z_{i}^{-\beta }}}\right]}^{\lambda -1}\left[\frac{z_{i}^{-\beta }e^{-2\alpha z_{i}^{-\beta }}\log(z_{i})}{{\left[1-\bar{\lambda }e^{-2\alpha z_{i}^{-\beta }}\right]}^{2}}\right]\right].$$

By equating them partial derivatives by zero and using R software to solve them.

### 5.3 Weighted least squares method

The method of weighted least squares introduced by [76], minimizes its function to get the estimates of the parameters, the weighted least square function is defined by,

(12) $$WLSE(\lambda ,\alpha ,\beta )=\sum_{i=1}^{n}\frac{{\left(n+1\right)}^{2}(n+2)}{i(n-i+1)}{\left[F(z_{\left(i\right)},\alpha ,\beta ,\lambda )-\frac{i}{n+1}\right]}^{2}=\sum_{i=1}^{n}\frac{{\left(n+1\right)}^{2}(n+2)}{i(n-i+1)}{\left[1-{\left[\frac{1-e^{-2\alpha z_{i}^{-\beta }}}{1-\bar{\lambda }e^{-2\alpha z_{i}^{-\beta }}}\right]}^{\lambda }-\frac{i}{n+1}\right]}^{2},$$

to minimize equation (12) by solving its partial derivative equations after equating them with zero, $\frac{\partial WLSE(\alpha ,\beta ,\lambda )}{\partial \alpha }=0$, $\frac{\partial WLSE(\alpha ,\beta ,\lambda )}{\partial \beta }=0$ and $\frac{\partial WLSE(\alpha ,\beta ,\lambda )}{\partial \lambda }=0$ using R software, the partial derivatives are:

$$\frac{\partial WLSE(\lambda ,\alpha ,\beta )}{\partial \lambda }=2\lambda \sum_{i=1}^{n}\frac{{\left(n+1\right)}^{2}(n+2)}{i(n-i+1)}\left[1-{\left[\frac{1-e^{-2\alpha z_{i}^{-\beta }}}{1-\bar{\lambda }e^{-2\alpha z_{i}^{-\beta }}}\right]}^{\lambda }-\frac{i}{n+1}\right]\frac{e^{-2\alpha z_{i}^{-\beta }}{\left[1-e^{-2\alpha z_{i}^{-\beta }}\right]}^{\lambda }}{{\left[1-\bar{\lambda }e^{-2\alpha z_{i}^{-\beta }}\right]}^{\lambda +1}},$$

$$\frac{\partial WLSE(\lambda ,\alpha ,\beta )}{\partial \alpha }=4\lambda^{2}\sum_{i=1}^{n}\frac{{\left(n+1\right)}^{2}(n+2)}{i(n-i+1)}\left[1-{\left[\frac{1-e^{-2\alpha z_{i}^{-\beta }}}{1-\bar{\lambda }e^{-2\alpha z_{i}^{-\beta }}}\right]}^{\lambda }-\frac{i}{n+1}\right]\left[-{\left[\frac{1-e^{-2\alpha z_{i}^{-\beta }}}{1-\bar{\lambda }e^{-2\alpha z_{i}^{-\beta }}}\right]}^{\lambda -1}\left[\frac{z_{i}^{-\beta }e^{-2\alpha z_{i}^{-\beta }}}{{\left[1-\bar{\lambda }e^{-2\alpha z_{i}^{-\beta }}\right]}^{2}}\right]\right],$$

and

$$\frac{\partial WLSE(\lambda ,\alpha ,\beta )}{\partial \beta }=4\alpha \lambda^{2}\sum_{i=1}^{n}\frac{{\left(n+1\right)}^{2}(n+2)}{i(n-i+1)}\left[1-{\left[\frac{1-e^{-2\alpha z_{i}^{-\beta }}}{1-\bar{\lambda }e^{-2\alpha z_{i}^{-\beta }}}\right]}^{\lambda }-\frac{i}{n+1}\right]\left[-{\left[\frac{1-e^{-2\alpha z_{i}^{-\beta }}}{1-\bar{\lambda }e^{-2\alpha z_{i}^{-\beta }}}\right]}^{\lambda -1}\left[\frac{z_{i}^{-\beta }e^{-2\alpha z_{i}^{-\beta }}\log(z_{i})}{{\left[1-\bar{\lambda }e^{-2\alpha z_{i}^{-\beta }}\right]}^{2}}\right]\right].$$

### 5.4 Anderson-Darling method

Anderson-Darling Method presented by [77], to get the estimates of *λ*, *α* and *β* we minimize the function with respect to the parameters, the function is:

$$A(\lambda ,\alpha ,\beta )=-n-\frac{1}{n}\sum_{i=1}^{n}(2i-1)\left[\log(F(z_{\left(i\right)},\lambda ,\alpha ,\beta ))+\log(1-F(z_{\left(n+i-1\right)},\lambda ,\alpha ,\beta ))\right]=-n-\frac{1}{n}\sum_{i=1}^{n}(2i-1)\left[\log\left(1-{\left[\frac{1-e^{-2\alpha z_{i}^{-\beta }}}{1-\bar{\lambda }e^{-2\alpha z_{i}^{-\beta }}}\right]}^{\lambda }\right)+\lambda \log\left(\frac{1-e^{-2\alpha z_{n+i-1}^{-\beta }}}{1-\bar{\lambda }e^{-2\alpha z_{n+i-1}^{-\beta }}}\right)\right].$$

Using the following abbreviations to simplify writing the partial equations

$$\eta_{i}=\frac{\lambda e^{-2\alpha z_{i}^{-\beta }}{\left[1-e^{-2\alpha z_{i}^{-\beta }}\right]}^{\lambda }}{{\left[1-\bar{\lambda }e^{-2\alpha z_{i}^{-\beta }}\right]}^{\lambda +1}},$$

$$\psi_{i}=\left[-{\left[\frac{1-e^{-2\alpha z_{i}^{-\beta }}}{1-\bar{\lambda }e^{-2\alpha z_{i}^{-\beta }}}\right]}^{\lambda -1}\left[\frac{2\lambda^{2}z_{i}^{-\beta }e^{-2\alpha z_{i}^{-\beta }}}{{\left[1-\bar{\lambda }e^{-2\alpha z_{i}^{-\beta }}\right]}^{2}}\right]\right],$$

and

$$\rho_{i}=\left[-{\left[\frac{1-e^{-2\alpha z_{i}^{-\beta }}}{1-\bar{\lambda }e^{-2\alpha z_{i}^{-\beta }}}\right]}^{\lambda -1}\left[\frac{2\alpha \lambda^{2}z_{i}^{-\beta }e^{-2\alpha z_{i}^{-\beta }}\log(z_{i})}{{\left[1-\bar{\lambda }e^{-2\alpha z_{i}^{-\beta }}\right]}^{2}}\right]\right].$$

The partial derivative with respect to the parameters *λ*, *α* and *β* are:

$$\frac{\partial A(\lambda ,\alpha ,\beta )}{\partial \lambda }=-\frac{1}{n}\sum_{i=1}^{n}(2i-1)\left[\frac{\eta_{i}}{F(z_{\left(i\right)},\lambda ,\alpha ,\beta ))}-\frac{\eta_{n+i-1}}{1-F(z_{\left(n+i-1\right)},\lambda ,\alpha ,\beta )}\right],$$

$$\frac{\partial A(\lambda ,\alpha ,\beta )}{\partial \alpha }=-\frac{1}{n}\sum_{i=1}^{n}(2i-1)\left[\frac{\psi_{i}}{F(z_{\left(i\right)},\lambda ,\alpha ,\beta ))}-\frac{\psi_{n+i-1}}{1-F(z_{\left(n+i-1\right)},\lambda ,\alpha ,\beta )}\right],$$

and

$$\frac{\partial A(\lambda ,\alpha ,\beta )}{\partial \beta }=-\frac{1}{n}\sum_{i=1}^{n}(2i-1)\left[\frac{\rho_{i}}{F(z_{\left(i\right)},\lambda ,\alpha ,\beta ))}-\frac{\rho_{n+i-1}}{1-F(z_{\left(n+i-1\right)},\lambda ,\alpha ,\beta )}\right].$$

By making the same steps done with the previous methods to get the estimates of *λ*, *α*, and *β* depending on R software, we will do that in the next section.

## 6 Simulation

In this section we do the numerical study using R software to find the optimum method of estimation from previous methods, to estimate (α,β,λ) of the NE_Fr, we generate random samples of different sizes of *n* = (25,50,75,100,125,150,175,200,225,250) from NE_Fr with replication 1000 times and for different initial values of (α,β,λ).

Cost-Effectiveness of Numerical Methods:

we used four methods of estimations: MLE, LSE, WLSE and ADE. The computational cost of MLE method is high, because it offers high accuracy and is robust for large samples. MLE needs iterative optimization method, which is the Nelder-Mead algorithm that requires more iterations. The computational complexity of MLE depends on the number of iterations required for convergence and the complexity of evaluating the likelihood function, and in our case MLE is complex function needs numerical methods to be solved. This complexity leads to longer runtime.

LSE and WLSE are simpler and their computational complexity is lower than MLE, as it depends only on sample size, so their computational cost and runtime are less than their corresponding in MLE. ADE is a modification of the LSE, its computational complexity and cost is slightly higher than LSE, so it needs runtime more than LSE, but still less that MLE runtime.

**Figure 4:**
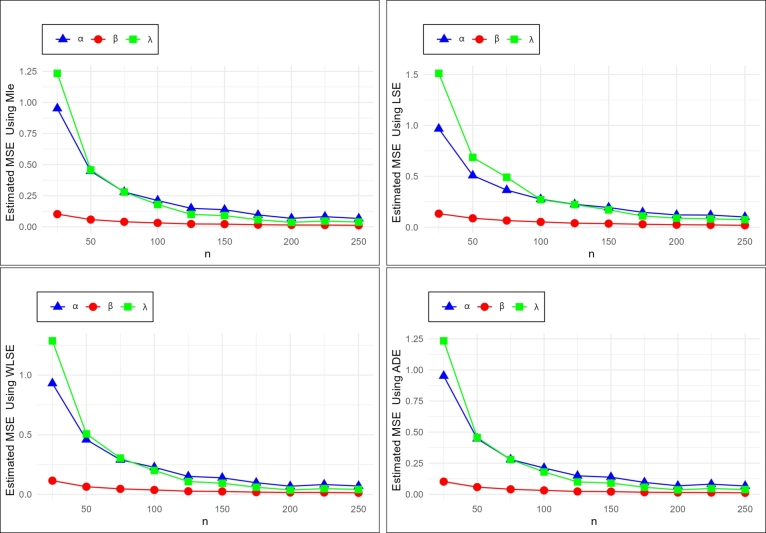
MSE for MLE, LSE, WLSE and ADE schemes in Table 5.

**Figure 5:**
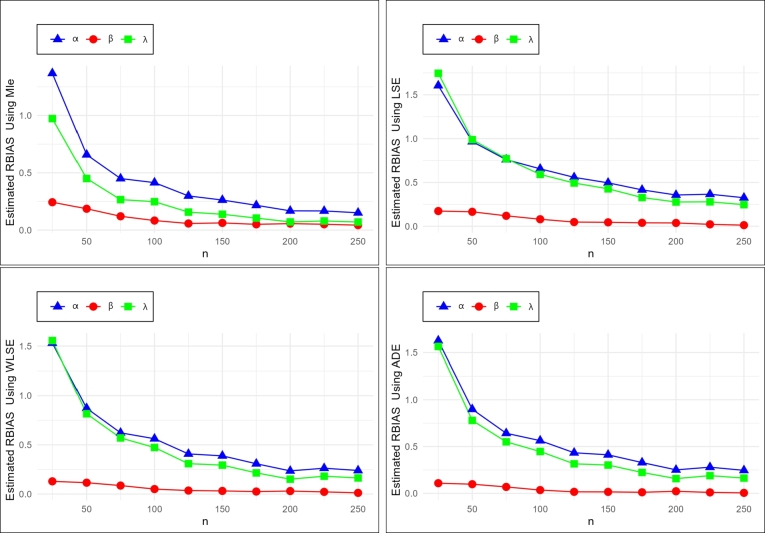
RBIAS for MLE, LSE, WLSE and ADE schemes in Table 5.

The estimates of the mean, RBias and mean square error (MSE) exist in Table 5, Table 6, Table 7, Table 8, Table 9, Table 10, the graph of MSE and RBIAS in Fig. 4 and Fig. 5 show:

• the estimated parameters (α,β,λ) goes closer to their initial values as *n* increases.
• the values of MSE and RBias go smaller as *n* increases.
• To compare between the used methods we need to have the ranks for all tables which are summarized in Table 11. The sum overall ranks for the MLE, LSE, WLSE, and ADE are 70, 240, 167, and 122.

**Table 11:** The ranks for all methods of estimation.

Parameters	n	MLE	LSE	WLSE	ADE	Parameters	n	MLE	LSE	WLSE	ADE
	25	1	4	2	3		25	2	4	3	1
	50	1	4	3	2		50	1	4	3	2
*α* = 0.3	75	1	4	3	2	*α* = 0.7	75	1	4	3	2
	100	1	4	3	2		100	1	4	3	2
	125	1	4	3	2		125	1	4	3	2
*β* = 0.4	150	1	4	3	2	*β* = 0.9	150	1	4	2.5	2.5
	175	1	4	3	2		175	1	4	3	2
	200	1	4	3	2		200	1	4	2	3
*λ* = 0.3	225	1	4	3	2	*λ* = 0.8	225	1	4	2.5	2.5
	250	1	4	3	2		250	1.5	4	1.5	3

	25	2	4	3	1		25	2	4	3	1
	50	1	4	3	2		50	1	4	3	2
*α* = 0.4	75	1	4	3	2	*α* = 0.9	75	1	4	3	2
	100	1	4	3	2		100	1	4	3	2
	125	1	4	3	2		125	1	4	2.5	2.5
*β* = 0.6	150	1	4	3	2	*β* = 1	150	1.5	4	3	1.5
	175	1	4	2	2		175	1	4	3	2
	200	1	4	2	3		200	2.5	4	2.5	1
*λ* = 0.8	225	1	4	2	3	*λ* = 0.9	225	1.5	4	1.5	3
	250	1	4	2	3		250	2	4	3	1

	25	3	4	2	1		25	1	4	3	2
	50	1	4	3	2		50	1	4	3	2
*α* = 0.5	75	1	4	3	2	*α* = 1.2	75	1	4	3	2
	100	1	4	3	2		100	1.5	4	3	1.5
	125	1	4	3	2		125	1.5	4	3	1.5
*β* = 0.8	150	1	4	3	2	*β* = 0.7	150	1	4	3	2
	175	1	4	3	2		175	1	4	3	2
	200	1	4	3	2		200	1	4	3	2
*λ* = 0.3	225	1	4	3	2	*λ* = 0.9	225	1	4	3	2
	250	1	4	2	3		250	1	4	3	2
• We can note that the MLE method is the preferable method to estimate α,β, and *λ*.

**Table 5:** Simulation results with initial values *α* = 0.3, *β* = 0.4 and *λ* = 0.3.

n	Estimate	MLE			LSE			WLSE			ADE		
		*α*	*β*	*λ*	*α*	*β*	*λ*	*α*	*β*	*λ*	*α*	*β*	*λ*
25	Mean	0.71046	0.49722	0.59181	0.78151	0.46955	0.82352	0.75929	0.45163	0.76673	0.78825	0.44343	0.76950
	RBIAS	1.36821	0.24304	0.97269	1.60503	0.17387	1.74506	1.53096	0.12908	1.55577	1.62751	0.10858	1.56498
	MSE	0.82357	0.11778	0.77558	0.96723	0.13330	1.51155	0.93069	0.11504	1.28690	0.95200	0.10205	1.23416

50	Mean	0.49729	0.47443	0.43498	0.58984	0.46610	0.59633	0.56164	0.44611	0.54479	0.56953	0.43914	0.53368
	RBIAS	0.65762	0.18608	0.44992	0.96613	0.16526	0.98777	0.87212	0.11529	0.81596	0.89844	0.09785	0.77894
	MSE	0.30486	0.06206	0.26628	0.50822	0.08800	0.68600	0.45873	0.06429	0.50676	0.44765	0.05814	0.45704

75	Mean	0.43491	0.44813	0.37967	0.52806	0.44780	0.53139	0.48669	0.43441	0.47101	0.49253	0.42750	0.46510
	RBIAS	0.44969	0.12033	0.26558	0.76019	0.11950	0.77131	0.62229	0.08603	0.57002	0.64176	0.06876	0.55035
	MSE	0.17308	0.03677	0.11208	0.36443	0.06553	0.49079	0.28993	0.04614	0.30386	0.28005	0.04035	0.27967

100	Mean	0.42421	0.43326	0.37432	0.49666	0.43216	0.47754	0.46869	0.42019	0.44195	0.46900	0.41406	0.43410
	RBIAS	0.41404	0.08315	0.24774	0.65555	0.08039	0.59181	0.56230	0.05046	0.47318	0.56334	0.03515	0.44699
	MSE	0.14258	0.02944	0.08600	0.27522	0.05197	0.27106	0.22688	0.03705	0.20070	0.21152	0.03145	0.17955

125	Mean	0.38941	0.42296	0.34700	0.46744	0.41923	0.44805	0.42230	0.41418	0.39261	0.43008	0.40654	0.39459
	RBIAS	0.29802	0.05741	0.15665	0.55814	0.04807	0.49351	0.40767	0.03546	0.30870	0.43359	0.01635	0.31530
	MSE	0.09525	0.01940	0.04466	0.22483	0.03959	0.22250	0.15087	0.02674	0.10779	0.14872	0.02304	0.09983

150	Mean	0.37865	0.42497	0.34139	0.44871	0.41811	0.42822	0.41651	0.41260	0.38812	0.42351	0.40632	0.39066
	RBIAS	0.26217	0.06244	0.13798	0.49569	0.04528	0.42742	0.38836	0.03150	0.29375	0.41169	0.01579	0.30221
	MSE	0.08142	0.01860	0.03678	0.19380	0.03562	0.17064	0.13888	0.02460	0.09332	0.13765	0.02173	0.09015

175	Mean	0.36487	0.42001	0.33139	0.42461	0.41583	0.39837	0.39238	0.41014	0.36448	0.39872	0.40460	0.36701
	RBIAS	0.21623	0.05003	0.10462	0.41538	0.03959	0.32789	0.30795	0.02535	0.21493	0.32908	0.01150	0.22335
	MSE	0.05822	0.01393	0.02494	0.14647	0.02908	0.11083	0.09780	0.01890	0.06016	0.09575	0.01671	0.05702

200	Mean	0.35034	0.42258	0.32128	0.40702	0.41523	0.38307	0.37061	0.41211	0.34532	0.37534	0.40895	0.34724
	RBIAS	0.16782	0.05646	0.07094	0.35674	0.03808	0.27689	0.23536	0.03027	0.15106	0.25114	0.02238	0.15747
	MSE	0.04558	0.01268	0.01835	0.12091	0.02467	0.08928	0.06862	0.01575	0.03680	0.06837	0.01452	0.03588

225	Mean	0.35009	0.42022	0.32375	0.40973	0.40890	0.38365	0.37873	0.40876	0.35438	0.38381	0.40407	0.35673
	RBIAS	0.16696	0.05056	0.07918	0.36576	0.02226	0.27883	0.26243	0.02189	0.18127	0.27938	0.01017	0.18912
	MSE	0.04948	0.01174	0.02128	0.11936	0.02265	0.08185	0.08243	0.01596	0.04806	0.08157	0.01411	0.04642

250	Mean	0.34527	0.41714	0.32109	0.39768	0.40514	0.37400	0.37188	0.40476	0.34905	0.37377	0.40213	0.34897
	RBIAS	0.15090	0.04286	0.07032	0.32560	0.01286	0.24665	0.23959	0.01191	0.16352	0.24590	0.00533	0.16324
	MSE	0.04348	0.00999	0.01922	0.10083	0.01863	0.07447	0.07053	0.01304	0.04155	0.06714	0.01194	0.03840

**Table 6:** Simulation results with initial values *α* = 0.4, *β* = 0.6 and *λ* = 0.8.

n	Estimate	MLE			LSE			WLSE			ADE		
		*α*	*β*	*λ*	*α*	*β*	*λ*	*α*	*β*	*λ*	*α*	*β*	*λ*
25	Mean	0.654435	1.057348	1.203207	0.796166	1.076253	1.899132	0.772247	1.006646	1.792069	0.760922	0.950503	1.652125
	RBIAS	0.636088	0.762246	0.504009	0.990415	0.793756	1.373914	0.930618	0.677743	1.240086	0.902305	0.584171	1.065157
	MSE	0.931083	1.027884	3.02671	1.050311	1.303301	8.425728	0.980053	1.086561	7.782507	0.894075	0.874343	6.211798

50	Mean	0.516302	0.854341	1.018923	0.715825	0.866756	1.551889	0.654612	0.80744	1.332318	0.650563	0.779244	1.30128
	RBIAS	0.290756	0.423901	0.273653	0.789563	0.444593	0.939861	0.63653	0.345733	0.665397	0.626408	0.29874	0.6266
	MSE	0.358455	0.401271	1.213213	0.69485	0.656784	4.125294	0.529718	0.465536	2.449896	0.50167	0.38157	2.186442

75	Mean	0.452225	0.819953	0.885913	0.621013	0.824105	1.245149	0.562257	0.780226	1.07883	0.56611	0.759011	1.079494
	RBIAS	0.130563	0.366588	0.107392	0.552534	0.373508	0.556437	0.405643	0.300377	0.348538	0.415276	0.265018	0.349367
	MSE	0.192272	0.306026	0.524442	0.439183	0.462836	1.6714	0.304212	0.334387	0.858842	0.296177	0.296217	0.819415

100	Mean	0.424952	0.801095	0.843802	0.604097	0.780491	1.208375	0.538476	0.757497	1.038413	0.537563	0.74287	1.036929
	RBIAS	0.062379	0.335158	0.054753	0.510243	0.300819	0.510469	0.346191	0.262496	0.298016	0.343907	0.238117	0.296161
	MSE	0.166873	0.22486	0.398481	0.406306	0.350434	1.599658	0.279745	0.257597	0.760563	0.271026	0.229799	0.778477

125	Mean	0.399496	0.773449	0.802548	0.578155	0.751288	1.133133	0.517699	0.724637	0.989848	0.515928	0.719716	0.987956
	RBIAS	0.001259	0.289082	0.003186	0.445388	0.252147	0.416416	0.294247	0.207729	0.237309	0.28982	0.199526	0.234945
	MSE	0.119073	0.166036	0.247741	0.333428	0.273751	1.073511	0.215995	0.191107	0.51442	0.213811	0.177463	0.515103

150	Mean	0.402428	0.750893	0.802681	0.52864	0.745649	1.027735	0.488377	0.719235	0.939723	0.492398	0.707904	0.943735
	RBIAS	0.006069	0.251488	0.003351	0.3216	0.242748	0.284669	0.220944	0.198725	0.174653	0.230995	0.17984	0.179668
	MSE	0.104425	0.13845	0.19965	0.253636	0.233476	0.6971	0.177696	0.164593	0.393731	0.172767	0.151244	0.382938

175	Mean	0.395459	0.732978	0.794822	0.529745	0.721353	1.027608	0.485613	0.696458	0.932767	0.487024	0.690264	0.936143
	RBIAS	0.011352	0.22163	0.006472	0.324363	0.202255	0.28451	0.214032	0.160764	0.165959	0.217559	0.15044	0.170179
	MSE	0.094284	0.105636	0.186602	0.242734	0.195646	0.655789	0.161252	0.132802	0.354522	0.160498	0.123429	0.363067

200	Mean	0.383514	0.734869	0.778156	0.523366	0.712918	1.008479	0.478636	0.694367	0.918877	0.482905	0.687091	0.925708
	RBIAS	0.041214	0.224782	0.027305	0.308416	0.188197	0.260599	0.196589	0.157279	0.148596	0.207263	0.145151	0.157134
	MSE	0.088435	0.105074	0.165542	0.230851	0.186666	0.576587	0.155081	0.130325	0.320422	0.155226	0.122008	0.324599

225	Mean	0.363608	0.731964	0.747316	0.500386	0.715673	0.964325	0.450782	0.697225	0.87311	0.454228	0.691469	0.878522
	RBIAS	0.09098	0.219939	0.065855	0.250964	0.192789	0.205406	0.126955	0.162042	0.091387	0.13557	0.152448	0.098152
	MSE	0.069703	0.086337	0.123891	0.196396	0.174621	0.460765	0.124621	0.112859	0.244691	0.124804	0.106599	0.24841

250	Mean	0.374095	0.711942	0.764751	0.502188	0.687047	0.969064	0.459074	0.670608	0.888596	0.462111	0.667466	0.893603
	RBIAS	0.064762	0.18657	0.044061	0.25547	0.145078	0.21133	0.147684	0.11768	0.110745	0.155277	0.112444	0.117004
	MSE	0.066538	0.069206	0.119783	0.18292	0.130475	0.442649	0.116751	0.084833	0.237246	0.11817	0.082039	0.244798

**Table 7:** Simulation results with initial values *α* = 0.5, *β* = 0.8 and *λ* = 0.3.

n	Estimate	MLE			LSE			WLSE			ADE		
		*α*	*β*	*λ*	*α*	*β*	*λ*	*α*	*β*	*λ*	*α*	*β*	*λ*
25	Mean	1.03912	1.03618	0.59240	1.00506	0.99628	0.78274	0.98717	0.94782	0.76239	1.03849	0.92726	0.74828
	RBIAS	1.07825	0.29522	0.97465	1.01012	0.24535	1.60915	0.97433	0.18478	1.54129	1.07697	0.15908	1.49428
	MSE	1.34482	0.51212	0.89222	1.21752	0.58477	1.37453	1.22050	0.47321	1.32898	1.21454	0.43902	1.20073

50	Mean	0.75457	0.90204	0.45272	0.86145	0.88304	0.64692	0.81353	0.85241	0.60094	0.82159	0.83383	0.58298
	RBIAS	0.50914	0.12754	0.50906	0.72291	0.10381	1.15639	0.62707	0.06551	1.00314	0.64318	0.04229	0.94326
	MSE	0.40525	0.22089	0.27346	0.66656	0.35082	0.73312	0.58728	0.25964	0.63353	0.55258	0.22495	0.53355

75	Mean	0.67766	0.87273	0.39532	0.76837	0.87418	0.53496	0.72485	0.83928	0.48825	0.72405	0.82890	0.47534
	RBIAS	0.35533	0.09092	0.31775	0.53674	0.09273	0.78320	0.44970	0.04910	0.62749	0.44811	0.03612	0.58448
	MSE	0.24380	0.14531	0.12232	0.43664	0.26702	0.41338	0.35776	0.18698	0.28962	0.33819	0.15763	0.26335

100	Mean	0.61604	0.87712	0.35736	0.69526	0.88303	0.46216	0.65704	0.85840	0.42371	0.66265	0.83628	0.41784
	RBIAS	0.23209	0.09640	0.19120	0.39052	0.10379	0.54054	0.31408	0.07300	0.41238	0.32531	0.04535	0.39279
	MSE	0.16879	0.10554	0.07080	0.31424	0.21125	0.26068	0.25379	0.14710	0.17514	0.24064	0.11579	0.15287

125	Mean	0.59554	0.86877	0.33897	0.65434	0.86296	0.42978	0.62685	0.84225	0.39476	0.63153	0.83412	0.39020
	RBIAS	0.19109	0.08596	0.12990	0.30868	0.07870	0.43261	0.25369	0.05281	0.31585	0.26307	0.04265	0.30067
	MSE	0.12494	0.09018	0.03679	0.25176	0.16573	0.19615	0.19796	0.11609	0.11604	0.18906	0.10108	0.10210

150	Mean	0.57920	0.86205	0.33247	0.63049	0.85917	0.39758	0.60758	0.83717	0.37391	0.61308	0.82877	0.37330
	RBIAS	0.15839	0.07756	0.10824	0.26098	0.07396	0.32526	0.21517	0.04647	0.24635	0.22615	0.03596	0.24435
	MSE	0.11005	0.07391	0.03555	0.19833	0.13785	0.12600	0.15880	0.09505	0.08302	0.15480	0.08396	0.07843

175	Mean	0.57920	0.84683	0.32999	0.63633	0.83530	0.39517	0.60544	0.82471	0.36526	0.61203	0.81670	0.36633
	RBIAS	0.15839	0.05853	0.09997	0.27265	0.04412	0.31722	0.21088	0.03089	0.21754	0.22406	0.02088	0.22108
	MSE	0.08603	0.06165	0.02474	0.17168	0.11730	0.10756	0.12750	0.08100	0.05928	0.12629	0.07306	0.05587

200	Mean	0.56720	0.84207	0.32271	0.61609	0.83070	0.37971	0.58743	0.82136	0.35203	0.59390	0.81309	0.35367
	RBIAS	0.13441	0.05258	0.07569	0.23219	0.03837	0.26571	0.17487	0.02670	0.17343	0.18780	0.01636	0.17890
	MSE	0.07142	0.05133	0.01855	0.14768	0.10398	0.08397	0.10577	0.06854	0.04667	0.10411	0.06102	0.04480

225	Mean	0.55646	0.85748	0.31612	0.60979	0.84487	0.37331	0.57737	0.83785	0.34359	0.58285	0.82897	0.34548
	RBIAS	0.11292	0.07185	0.05374	0.21958	0.05608	0.24436	0.15474	0.04731	0.14530	0.16570	0.03621	0.15161
	MSE	0.06986	0.05337	0.01818	0.14516	0.10815	0.07934	0.10348	0.07076	0.04039	0.10205	0.06256	0.03955

250	Mean	0.57171	0.82162	0.32801	0.62356	0.80355	0.38585	0.59664	0.79793	0.35862	0.60193	0.79149	0.36022
	RBIAS	0.14341	0.02702	0.09336	0.24712	0.00443	0.28615	0.19328	0.00259	0.19539	0.20386	0.01063	0.20074
	MSE	0.06330	0.04027	0.01723	0.13896	0.08321	0.08031	0.09854	0.05568	0.04220	0.09787	0.05100	0.04101

**Table 8:** Simulation results with initial values *α* = 0.7, *β* = 0.9 and *λ* = 0.8.

n	Estimate	MLE			LSE			WLSE			ADE		
		*α*	*β*	*λ*	*α*	*β*	*λ*	*α*	*β*	*λ*	*α*	*β*	*λ*
25	Mean	0.96173	1.56652	1.29039	1.03963	1.61577	1.88992	1.01614	1.51826	1.74833	1.05637	1.40837	1.67217
	RBIAS	0.37390	0.74058	0.61299	0.48518	0.79530	1.36240	0.45163	0.68695	1.18541	0.50911	0.56485	1.09021
	MSE	1.21059	2.38210	3.90688	1.18305	3.22398	7.64254	1.14204	2.63499	7.10429	1.09146	2.12931	5.48859

50	Mean	0.74215	1.33574	0.97111	0.86877	1.42740	1.34976	0.84656	1.30215	1.22612	0.85574	1.24388	1.18680
	RBIAS	0.06021	0.48415	0.21389	0.24110	0.58600	0.68720	0.20937	0.44684	0.53264	0.22249	0.38209	0.48350
	MSE	0.42374	0.99976	1.00587	0.69858	1.79799	2.83154	0.58353	1.23882	1.89272	0.53379	1.00315	1.48478

75	Mean	0.73176	1.22280	0.91262	0.87631	1.21557	1.29334	0.84866	1.14211	1.14310	0.86224	1.10303	1.14982
	RBIAS	0.04537	0.35866	0.14078	0.25187	0.35064	0.61668	0.21237	0.26901	0.42888	0.23177	0.22559	0.43727
	MSE	0.32422	0.67622	0.59660	0.58994	1.00968	2.04057	0.46370	0.71035	1.13534	0.44269	0.59987	1.14595

100	Mean	0.69018	1.19940	0.85258	0.79043	1.24742	1.11260	0.76277	1.17403	0.99742	0.77656	1.14205	1.00440
	RBIAS	0.01403	0.33267	0.06573	0.12918	0.38602	0.39075	0.08967	0.30447	0.24677	0.10938	0.26895	0.25550
	MSE	0.25938	0.53957	0.40692	0.46994	0.92515	1.29997	0.36158	0.63664	0.69999	0.35264	0.56311	0.69186

125	Mean	0.68015	1.13861	0.81926	0.80132	1.14684	1.07272	0.77105	1.08931	0.97058	0.77963	1.07121	0.97305
	RBIAS	0.02836	0.26512	0.02407	0.14474	0.27427	0.34090	0.10151	0.21035	0.21323	0.11376	0.19023	0.21632
	MSE	0.19874	0.36120	0.26885	0.39967	0.64827	0.88358	0.29551	0.43699	0.49282	0.28737	0.39351	0.48288

150	Mean	0.65875	1.11975	0.78821	0.79200	1.12025	1.04324	0.75655	1.07255	0.93899	0.76336	1.05736	0.94513
	RBIAS	0.05893	0.24417	0.01474	0.13142	0.24473	0.30405	0.08079	0.19173	0.17374	0.09051	0.17484	0.18142
	MSE	0.15937	0.29299	0.19874	0.35758	0.57249	0.72888	0.24997	0.39298	0.39327	0.24787	0.35200	0.40778

175	Mean	0.63550	1.12980	0.75901	0.77347	1.09216	0.99776	0.72798	1.06701	0.89459	0.73192	1.05824	0.89518
	RBIAS	0.09214	0.25533	0.05123	0.10495	0.21351	0.24720	0.03997	0.18557	0.11824	0.04560	0.17582	0.11898
	MSE	0.14983	0.26654	0.16125	0.31337	0.46553	0.57255	0.22254	0.31404	0.30737	0.21783	0.29209	0.30248

200	Mean	0.64506	1.10499	0.76920	0.77897	1.07494	1.00470	0.74044	1.04376	0.90725	0.74463	1.03847	0.91209
	RBIAS	0.07849	0.22776	0.03850	0.11282	0.19438	0.25588	0.05777	0.15973	0.13406	0.06376	0.15386	0.14011
	MSE	0.14550	0.22940	0.15825	0.31301	0.42023	0.59759	0.21945	0.28410	0.31263	0.22083	0.26974	0.32621

225	Mean	0.64699	1.07388	0.76750	0.77917	1.03804	0.99018	0.74297	1.00959	0.90109	0.74986	1.00165	0.90676
	RBIAS	0.07574	0.19320	0.04063	0.11310	0.15337	0.23772	0.06139	0.12176	0.12636	0.07123	0.11295	0.13345
	MSE	0.12167	0.16867	0.13241	0.27692	0.32807	0.49293	0.19019	0.21122	0.26228	0.18808	0.20161	0.26433

250	Mean	0.62793	1.08848	0.74873	0.73993	1.07696	0.94075	0.70882	1.04208	0.86218	0.71574	1.03241	0.86961
	RBIAS	0.10295	0.20942	0.06409	0.05705	0.19662	0.17594	0.01259	0.15786	0.07772	0.02248	0.14712	0.08701
	MSE	0.11479	0.17149	0.11963	0.25812	0.35773	0.43917	0.17487	0.22966	0.23089	0.17528	0.21415	0.23940

**Table 9:** Simulation results with initial values *α* = 0.9, *β* = 1 and *λ* = 0.9.

n	Estimate	MLE			LSE			WLSE			ADE		
		*α*	*β*	*λ*	*α*	*β*	*λ*	*α*	*β*	*λ*	*α*	*β*	*λ*
25	Mean	1.060981	1.897805	1.414027	1.142937	1.869604	2.023289	1.134966	1.756417	1.892799	1.161763	1.69624	1.800018
	RBIAS	0.178868	0.897805	0.571141	0.26993	0.869604	1.248099	0.261073	0.756417	1.10311	0.290848	0.69624	1.00002
	MSE	1.065093	3.858234	4.258614	1.109907	4.498763	8.628471	1.056504	3.692777	7.26593	1.04153	3.32516	6.170579

50	Mean	0.89661	1.581015	1.106329	1.057605	1.613653	1.643429	1.019318	1.50455	1.413647	1.026691	1.440835	1.37954
	RBIAS	0.003767	0.581015	0.229255	0.175117	0.613653	0.826032	0.132576	0.50455	0.570719	0.140767	0.440835	0.532823
	MSE	0.499232	1.733185	1.583178	0.81023	2.645495	4.686683	0.655161	1.957729	2.673134	0.617822	1.575976	2.432688

75	Mean	0.84762	1.416695	0.961233	0.980969	1.462704	1.330145	0.954752	1.352547	1.178163	0.959908	1.313802	1.16302
	RBIAS	0.0582	0.416695	0.068037	0.089966	0.462704	0.477938	0.060835	0.352547	0.30907	0.066564	0.313802	0.292245
	MSE	0.340432	0.934999	0.606535	0.593631	1.685187	1.926793	0.459425	1.136356	1.029385	0.437959	0.913489	0.977629

100	Mean	0.842143	1.348274	0.913582	0.964838	1.395476	1.242162	0.936134	1.3038	1.103439	0.942982	1.272225	1.098059
	RBIAS	0.064285	0.348274	0.015091	0.072042	0.395476	0.38018	0.040149	0.3038	0.226044	0.047758	0.272225	0.220066
	MSE	0.266238	0.697346	0.389125	0.505723	1.373807	1.504365	0.380562	0.879113	0.777821	0.365287	0.741606	0.733596

125	Mean	0.833654	1.299215	0.887953	0.951709	1.316576	1.177166	0.919572	1.252589	1.051945	0.937505	1.218593	1.06504
	RBIAS	0.073717	0.299215	0.013386	0.057455	0.316576	0.307962	0.021747	0.252589	0.168828	0.041672	0.218593	0.183378
	MSE	0.227134	0.530627	0.277341	0.433162	0.954177	1.042948	0.321041	0.648727	0.53971	0.313591	0.558468	0.542148

150	Mean	0.836572	1.262552	0.883087	0.911062	1.311178	1.086881	0.908909	1.227384	1.017527	0.910835	1.216036	1.010607
	RBIAS	0.070476	0.262552	0.018792	0.012291	0.311178	0.207646	0.009899	0.227384	0.130586	0.012039	0.216036	0.122897
	MSE	0.20855	0.391522	0.241147	0.3789	0.796845	0.784811	0.286098	0.514515	0.460932	0.277908	0.463878	0.419064

175	Mean	0.824744	1.249814	0.861621	0.929693	1.264377	1.089038	0.896823	1.214673	0.988201	0.908834	1.196809	0.996085
	RBIAS	0.083617	0.249814	0.042643	0.032992	0.264377	0.210042	0.00353	0.214673	0.098001	0.009816	0.196809	0.106761
	MSE	0.180623	0.332253	0.202436	0.353193	0.703408	0.65515	0.256072	0.454038	0.363923	0.250044	0.416624	0.358991

200	Mean	0.788334	1.266376	0.827599	0.889599	1.239674	1.032196	0.871194	1.19614	0.957246	0.875181	1.18929	0.959129
	RBIAS	0.124073	0.266376	0.080446	0.011557	0.239674	0.146884	0.032006	0.19614	0.063607	0.027577	0.18929	0.065699
	MSE	0.165197	0.326243	0.167827	0.301844	0.547068	0.515948	0.221827	0.369808	0.296776	0.218493	0.347596	0.299094

225	Mean	0.822116	1.212406	0.855418	0.928906	1.189353	1.069825	0.913914	1.140744	0.993449	0.922658	1.129999	1.003377
	RBIAS	0.086538	0.212406	0.049535	0.032118	0.189353	0.188695	0.01546	0.140744	0.103832	0.025175	0.129999	0.114863
	MSE	0.152919	0.250754	0.157613	0.301773	0.485328	0.530224	0.21392	0.310973	0.285516	0.214159	0.291125	0.301415

250	Mean	0.814844	1.211012	0.843399	0.921108	1.189433	1.044137	0.891553	1.16235	0.961917	0.898983	1.149842	0.966265
	RBIAS	0.094618	0.211012	0.06289	0.023454	0.189433	0.160152	0.009386	0.16235	0.068796	0.001131	0.149842	0.073628
	MSE	0.137366	0.229445	0.137511	0.276154	0.458309	0.45165	0.197423	0.305081	0.253204	0.192319	0.279519	0.249577

**Table 10:** Simulation results with initial values *α* = 1.2, *β* = 0.7 and *λ* = 0.9.

n	Estimate	MLE			LSE			WLSE			ADE		
		*α*	*β*	*λ*	*α*	*β*	*λ*	*α*	*β*	*λ*	*α*	*β*	*λ*
25	Mean	1.36570	1.37255	1.38020	1.40082	1.43351	1.89309	1.37940	1.29307	1.79728	1.41916	1.23442	1.66564
	RBIAS	0.13808	0.96079	0.53355	0.16735	1.04787	1.10344	0.14950	0.84725	0.99698	0.18264	0.76346	0.85071
	MSE	1.39409	2.00875	4.24777	1.80613	2.65276	7.60596	1.40496	1.96522	6.83765	1.64739	1.70585	5.15765

50	Mean	1.18445	1.05522	1.11366	1.26348	1.12787	1.53315	1.23988	1.04946	1.36196	1.27124	0.98692	1.33082
	RBIAS	0.01296	0.50745	0.23740	0.05290	0.61124	0.70350	0.03323	0.49923	0.51329	0.05936	0.40989	0.47869
	MSE	0.55375	0.69008	1.59253	0.82585	1.17143	3.59094	0.70951	0.85838	2.24504	0.65768	0.67661	1.95200

75	Mean	1.18794	0.96273	1.05634	1.26008	0.99582	1.38982	1.23941	0.93263	1.24439	1.26971	0.88790	1.24758
	RBIAS	0.01005	0.37533	0.17372	0.05007	0.42260	0.54424	0.03284	0.33233	0.38266	0.05809	0.26842	0.38620
	MSE	0.43946	0.49255	0.82993	0.67099	0.77637	2.14358	0.54972	0.54114	1.35063	0.52232	0.43885	1.27691

100	Mean	1.11622	0.94999	0.93017	1.20523	0.95602	1.21746	1.17166	0.91647	1.07880	1.19630	0.89189	1.08668
	RBIAS	0.06981	0.35713	0.03352	0.00436	0.36574	0.35274	0.02362	0.30925	0.19867	0.00309	0.27413	0.20742
	MSE	0.34103	0.34873	0.40618	0.52347	0.56449	1.35849	0.42140	0.39939	0.68750	0.40689	0.35027	0.66221

125	Mean	1.13486	0.89280	0.92552	1.18928	0.92864	1.14889	1.17953	0.87862	1.05127	1.19417	0.86065	1.05166
	RBIAS	0.05428	0.27544	0.02836	0.00893	0.32662	0.27654	0.01705	0.25517	0.16808	0.00486	0.22950	0.16851
	MSE	0.27947	0.24499	0.31103	0.46164	0.45739	0.93064	0.36297	0.31072	0.53410	0.34850	0.27439	0.51239

150	Mean	1.14508	0.86531	0.91402	1.18839	0.91968	1.11265	1.17674	0.87108	1.01943	1.19591	0.84770	1.02678
	RBIAS	0.04576	0.23616	0.01557	0.00967	0.31383	0.23628	0.01938	0.24440	0.13270	0.00340	0.21100	0.14087
	MSE	0.23335	0.19783	0.24559	0.41546	0.43063	0.75289	0.32183	0.28059	0.42896	0.30596	0.24222	0.41628

175	Mean	1.15146	0.84221	0.92166	1.16700	0.90176	1.07481	1.17177	0.85106	1.00498	1.18988	0.83210	1.01493
	RBIAS	0.04045	0.20316	0.02406	0.02750	0.28823	0.19424	0.02352	0.21580	0.11665	0.00843	0.18872	0.12770
	MSE	0.22495	0.16951	0.22165	0.39324	0.34735	0.67031	0.30098	0.23638	0.37631	0.28947	0.20892	0.36958

200	Mean	1.15696	0.81956	0.91502	1.17930	0.86153	1.05834	1.17748	0.82441	0.99067	1.19556	0.80439	1.00185
	RBIAS	0.03587	0.17080	0.01669	0.01725	0.23075	0.17593	0.01877	0.17773	0.10074	0.00370	0.14913	0.11317
	MSE	0.19083	0.12530	0.19529	0.34635	0.26878	0.54479	0.26077	0.18104	0.32242	0.25030	0.15510	0.31781

225	Mean	1.17153	0.79650	0.92930	1.18354	0.83829	1.06141	1.18898	0.79806	1.00176	1.20849	0.78235	1.01782
	RBIAS	0.02372	0.13786	0.03256	0.01372	0.19756	0.17934	0.00918	0.14009	0.11307	0.00708	0.11764	0.13091
	MSE	0.17471	0.10875	0.17484	0.31494	0.24489	0.50873	0.23623	0.15824	0.29973	0.23093	0.14289	0.30659

250	Mean	1.15372	0.80649	0.90074	1.17998	0.83598	1.03276	1.17638	0.80719	0.97370	1.19010	0.79513	0.98306
	RBIAS	0.03857	0.15212	0.00082	0.01669	0.19426	0.14751	0.01969	0.15313	0.08189	0.00825	0.13589	0.09229
	MSE	0.16210	0.10441	0.15016	0.30120	0.22254	0.42626	0.22768	0.15182	0.25987	0.22066	0.13838	0.26048

## 7 Applications

No one distribution can be considered the best for characterizing all sorts of data, despite the fact that there are several bounded distributions in the literature. In this part, three real-world healthcare data sets are statistically analyzed using the NE_Fr lifespan model. The first data set, designated as cancer data, shows the number of months that 128 individuals with bladder cancer went into remission. The COVID-19 data set represents the number of confirmed cases of COVID-19 (per day) in Pakistan. The third data set that the World Health Organization (WHO) has made available, COVID-19, displays the total number of fatalities reported globally in the previous 24 hours. Specifically, our goal is to assess how well the NE_Fr model fits in comparison to other Fr model generalizations NHL_Fr, B_Fr, EG_Fr, HL_Fr, E_Fr, Ga_Fr, TL_Fr, MO_Fr, and Fr models. These densities' positive real integers serve as the parameters.

The technique of maximum likelihood estimation is used to estimate the unknown parameters. When comparing datasets, we evaluate several criteria to determine suitability. There are several types of information criteria, such as the minus log-likelihood (*τ*1), the Akaike information criterion (*τ*2), correct Akaike information criterion (*τ*3), the Bayesian information criterion (*τ*4), Hannan-Quinn Akaike information criterion (*τ*5), the Cramér-von Mises (*τ*6) p-value, the Kolmogrov-Smirnov (*τ*7) p-value and the Anderson-Darling (*τ*8) p-value. The NE_Fr model achieves the least values for each criterion (except PV(*τ*7) with the highest value), suggesting that it offers the best match.

### 7.1 The first dataset

The dataset consists of the lengths of 128 bladder cancer patients' remissions (measured in months), taken from a randomly chosen sample [78]. In order to maintain transparency, the data are: 21.73, 4.4, 7.93, 7.39, 8.26, 4.34, 8.37, 3.48, 7.63, 2.83, 9.74, 3.25, 0.81, 10.66, 11.98, 14.76, 5.32, 0.5, 3.57, 13.11, 34.26, 1.26, 1.19, 2.02, 4.5, 0.51, 7.09, 7.28, 2.23, 2.69, 4.18, 26.31, 2.62, 12.02, 2.54, 0.2, 3.31, 5.49, 4.33, 5.32, 0.08, 1.46, 25.74, 5.62, 6.76, 7.87, 5.85, 7.62, 20.28, 5.41, 2.07, 5.17, 3.82, 17.14, 16.62, 22.69, 11.64, 0.4, 14.24, 4.23, 6.97, 3.02, 8.53, 11.25, 15.96, 6.25, 5.06, 4.51, 3.88, 13.29, 3.36, 10.34, 2.75, 2.64, 9.47, 5.41, 1.35, 6.94, 0.9, 2.09, 11.79, 17.36, 7.32, 32.15, 5.71, 9.02, 13.8, 10.06, 1.05, 19.13, 79.05, 6.93, 14.83, 3.52, 17.12, 5.09, 8.65, 2.69, 2.26, 12.03, 3.64, 7.66, 36.66, 1.76, 23.63, 12.07, 2.02, 3.36, 10.75, 9.22, 2.46, 14.77, 25.82, 4.87, 6.54, 12.63, 8.66, 5.34, 4.98, 1.4, 7.26, 3.7, 46.12, 18.1, 43.01, 2.87, 7.59, 4.26.

Table 12 presents several descriptive statistics of this dataset. The skewness and kurtosis suggest that the data follows an exponential distribution with a reversed-J shape.

**Table 12:** The descriptive statistics for data set1.

	dataset 1
n	128
Mean	9.37
Median	6.4
SD	10.51
Skewness	3.29
Kurtosis	15.48
Minimum	0.08
Maximum	79.05

**Figure 6:**
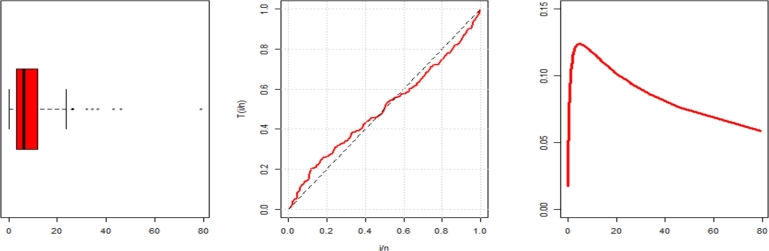
Box and TTT plot for data1.

**Figure 7:**
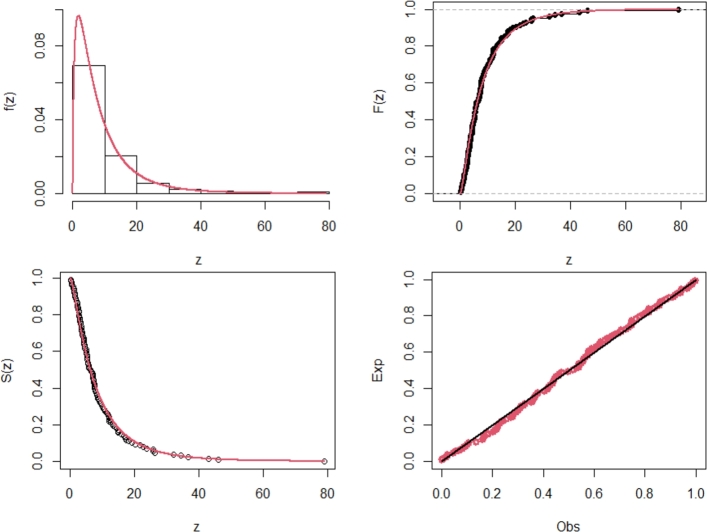
Estimated pdf, cdf, ccdf and PPlot plots of data1.

Fig. 6 displays the TTT plot in conjunction with the fitted hazard rate function. The TTT plot indicates a potential hrf pattern that alternates between growing and dropping, allowing for the fitting of the NE_Fr model on this dataset. *τ*1, *τ*2, *τ*3, *τ*4, *τ*5, *τ*6, *τ*7, and *τ*8 are shown in Table 14. They show the maximum likelihood estimators (with standard errors) in Table 13. For a better understanding, Fig. 7 of the NE_Fr model shows pdf, cdf, the complementary cumulative distribution function (ccdf), and the probability-probability (PP) plots. The figure displays excellent correlations for the NE_Fr model.

**Table 13:** Measures of fitting for data set1.

Model	Estimates							
	$\overset{ˆ}{\alpha }$	SE($\overset{ˆ}{\alpha }$)	$\overset{ˆ}{\beta }$	SE($\overset{ˆ}{\beta }$)	$\overset{ˆ}{\lambda }$	SE($\overset{ˆ}{\lambda }$)	$\overset{ˆ}{\theta }$	SE($\overset{ˆ}{\theta }$)
NE_Fr	4.847	1.069	0.154	0.052	32.157	36.035		
NHL_Fr	3.084	0.623	0.224	0.065	61.172	8.095		
B_Fr	0.739	0.420	62.050	47.844	0.269	0.076	8.036	2.169
EG_Fr	39.797	24.294	0.769	0.391	7.353	1.770	0.294	0.073
HL_Fr	83.554	10.822	6.458	1.270	0.217	0.065		
E_Fr	167.967	83.203	7.920	1.070	0.201	0.041		
Ga_Fr	10.081	0.335	0.002	0.000	2.144	0.120		
TL_Fr	0.769	0.450	4.128	1.968	0.617	0.053		
Mo_Fr	19.726	10.174	0.431	0.167	1.253	0.097		
Fr	2.432	0.219	0.752	0.042

**Table 14:** Measures of fitting for data set1.

Model	*τ*1	*τ*2	*τ*3	*τ*4	*τ*5	*τ*6	*τ*7	*τ*8
NE_Fr	821.502	827.502	827.696	836.058	830.978	0.043	0.918	0.282
NHL_Fr	821.807	827.807	828.001	836.363	831.283	0.046	0.877	0.308
B_Fr	823.823	831.823	832.148	843.234	836.458	0.060	0.852	0.413
EG_Fr	824.926	832.926	833.251	844.334	837.561	0.068	0.829	0.473
HL_Fr	821.969	827.969	828.163	836.418	831.445	0.051	0.869	0.317
E_Fr	822.724	828.724	828.918	837.280	832.200	0.052	0.807	0.358
Ga_Fr	873.990	879.990	880.184	888.555	883.466	0.556	0.016	3.461
TL_Fr	862.189	868.189	868.383	876.745	871.665	0.485	0.072	2.888
Mo_Fr	847.679	853.679	853.873	862.235	857.155	0.215	0.178	1.442
Fr	888.002	892.002	892.098	897.706	894.320	0.744	0.013	4.546

### 7.2 The second dataset

Recently, [79] conducted a study on the daily confirmed cases of COVID-19 in Pakistan, spanning from March to April 2020, covering 36 days. To ensure transparency, the following data points are provided: 35, 102, 38, 96, 204, 264, 663, 105, 25, 90, 67, 87, 172, 19, 69, 120, 26, 181, 627, 190, 111, 40, 163, 228, 3, 283, 155, 2, 68, 36, 192, 4, 4, 2, 199, 24.

**Figure 8:**
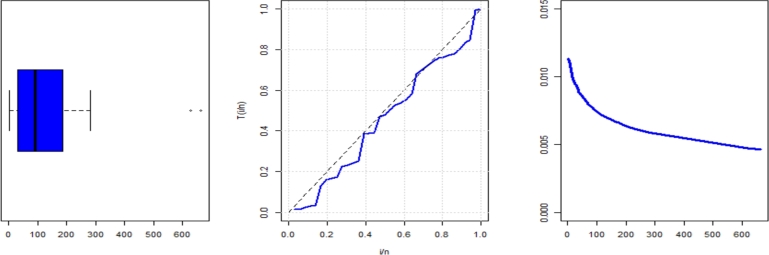
Box and TTT plot for data2.

The statistical characteristics of this data are shown in Table 15. In Fig. 8, the TTT plot shows a declining hrf, which is consistent with the predicted hrf of NE_Fr. *τ*1, *τ*2, *τ*3, *τ*4, *τ*5, *τ*6, *τ*7, and *τ*8 are shown in Table 17. They show The MLEs (with SE) in Table 16.

**Table 15:** The descriptive statistics for data set2.

	dataset 2
n	36
Mean	130.39
Median	93
SD	149.7
Skewness	2.27
Kurtosis	5.46
Minimum	2
Maximum	663

**Table 16:** Measures of fitting for data set2.

Model	Estimates							
	$\overset{ˆ}{\alpha }$	SE($\overset{ˆ}{\alpha }$)	$\overset{ˆ}{\beta }$	SE($\overset{ˆ}{\beta }$)	$\overset{ˆ}{\lambda }$	SE($\overset{ˆ}{\lambda }$)	$\overset{ˆ}{\theta }$	SE($\overset{ˆ}{\theta }$)
NE_Fr	8.826	1.043	0.064	0.012	663.117	662.566		
NHL_Fr	4.396	0.723	0.161	0.048	83.735	16.962		
B_Fr	0.262	0.202	76.639	24.821	0.294	0.102	25.207	17.063
EG_Fr	119.959	88.936	0.301	0.191	22.186	11.611	0.272	0.102
HL_Fr	8.843	1.408	0.161	0.049	91.797	24.633		
E_Fr	76.638	44.282	9.755	1.331	0.173	0.041		
Ga_Fr	15.330	1.853	0.003	0.002	2.276	0.377		
TL_Fr	0.407	0.216	17.291	7.654	0.502	0.083		
Mo_Fr	150.492	87.382	1.149	0.412	1.200	0.178		
Fr	7.145	1.702	0.593	0.070

**Table 17:** Measures of fitting for data set2.

Model	*τ*1	*τ*2	*τ*3	*τ*4	*τ*5	*τ*6	*τ*7	*τ*8
NE_Fr	422.087	428.087	428.837	432.837	428.837	0.079	0.852	0.571
NHL_Fr	422.632	428.632	429.382	433.382	430.290	0.094	0.780	0.662
B_Fr	422.891	430.891	432.181	437.225	433.102	0.113	0.490	0.773
EG_Fr	422.544	430.544	431.834	436.878	432.755	0.105	0.540	0.726
HL_Fr	423.869	429.869	430.619	434.619	431.527	0.105	0.720	0.693
E_Fr	424.964	430.964	431.714	435.714	432.622	0.138	0.510	0.931
Ga_Fr	434.435	440.435	441.185	445.185	442.093	0.314	0.140	1.970
TL_Fr	435.393	441.393	442.143	446.143	443.051	0.326	0.150	2.039
Mo_Fr	427.552	433.552	434.302	438.303	435.210	0.164	0.750	1.093
Fr	441.051	445.051	445.415	448.218	446.156	0.418	0.140	2.540

**Figure 9:**
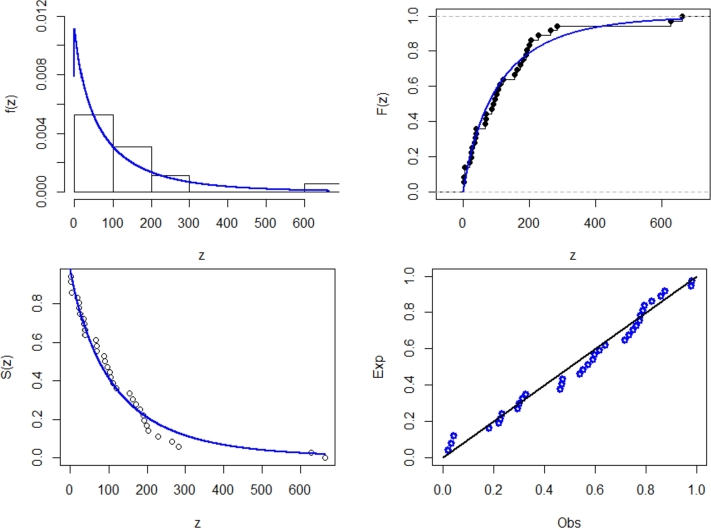
Estimated pdf, cdf, ccdf and PPlot plots of data2.

According to the GoF measurements, the NE_Fr model is much better than its rivals. There are four plots in Fig. 9 of the NE_Fr model: pdf, cdf, ccdf, and PP plots.

### 7.3 The third dataset

**Figure 10:**
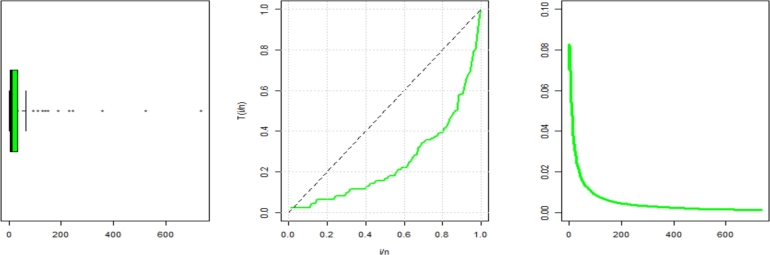
Box and TTT plot for data3.

**Figure 11:**
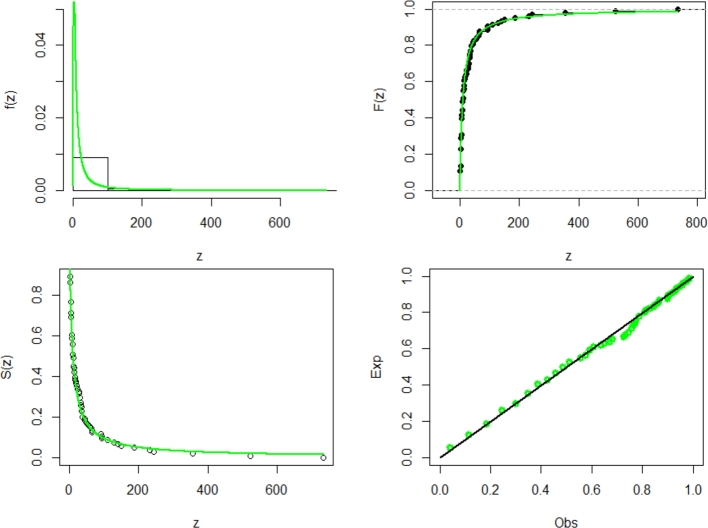
Estimated pdf, cdf, ccdf and PPlot plots of data3.

On October 26, 2021, the World Health Organization (WHO) was informed about COVID-19-related fatalities. The third data set includes the global fatalities documented during October 26, 2021. The data consists of the following numbers: 6, 15, 57, 1, 4, 66, 1, 6, 2, 14, 6, 4, 35, 6, 3, 5, 187, 6, 92, 2, 21, 3, 32, 30, 5, 110, 1, 3, 3, 35, 14, 128, 6, 34, 1, 1, 16, 6, 3, 9, 10, 7, 1, 38, 6, 9, 43, 27, 18, 734, 93, 33, 22, 6, 140, 4, 2, 356, 9, 15, 523, 232, 3, 1, 1, 38, 19, 9, 3, 11, 32, 1, 11, 11, 48, 38, 3, 13, 8, 4, 22, 15, 28, 10, 7, 8, 54, 4, 65, 243, 62, 3, 30, 8, 4, 11, 1, 44, 32, 149, 94, 3, 1, 9. Additionally, Table 18 presents the descriptive statistics about the COVID-2 data. TTT graph corresponds closely to the predicted HRF of NE_Fr, as shown in Fig. 10. They show The MLEs (with SE) in Table 19. *τ*1, *τ*2, *τ*3, *τ*4, *τ*5, *τ*6, *τ*7, and *τ*8, are shown in Table 20. The GoF measurements demonstrate the superiority of the NE_FR model compared to its rivals. pdf, cdf, ccdf, and PP plots are shown in a graph form in Fig. 11 of the NE_Fr model.

**Table 18:** The descriptive statistics for data set3.

	dataset 3
n	104
Mean	94.64
Median	10
SD	538.86
Skewness	9.61
Kurtosis	93.08
Minimum	1
Maximum	734

**Table 19:** Measures of fitting for data set3.

Model	Estimates							
	$\overset{ˆ}{\alpha }$	SE($\overset{ˆ}{\alpha }$)	$\overset{ˆ}{\beta }$	SE($\overset{ˆ}{\beta }$)	$\overset{ˆ}{\lambda }$	SE($\overset{ˆ}{\lambda }$)	$\overset{ˆ}{\theta }$	SE($\overset{ˆ}{\theta }$)
NE_Fr	2.621	0.456	0.339	0.137	2.745	1.51		
NHL_Fr	1.488	0.204	0.629	0.174	1.224	0.521		
B_Fr	0.109	0.176	1.725	0.764	0.634	0.101	31.791	5.098
EG_Fr	3.222	1.074	7.6	5.135	1.295	0.264	0.304	0.098
HL_Fr	1.143	0.614	2.883	0.505	0.76	0.264		
E_Fr	3.954	0.536	0.523	0.153	1.831	1.052		
Ga_Fr	7.293	4.273	0.043	0.128	1.631	0.537		
TL_Fr	0.154	0.088	22.46	14.567	0.617	0.117		
Mo_Fr	3.188	2.363	2.301	0.864	0.896	0.121		
Fr	3.552	0.411	0.717	0.054

**Table 20:** Measures of fitting for data set3.

Model	*τ*1	*τ*2	*τ*3	*τ*4	*τ*5	*τ*6	*τ*7	*τ*8
NE_Fr	902.057	908.057	915.990	908.297	911.271	0.050	0.743	0.454
NHL_Fr	930.506	936.506	944.460	936.746	939.720	0.053	0.692	0.526
B_Fr	929.733	937.733	948.349	938.137	942.018	0.058	0.671	0.558
EG_Fr	930.450	938.450	949.116	938.854	942.735	0.059	0.667	0.536
HL_Fr	937.709	943.709	945.671	943.949	946.923	0.056	0.661	0.591
E_Fr	932.514	938.514	945.538	938.754	941.728	0.067	0.680	0.685
Ga_Fr	947.546	953.546	958.507	953.786	956.760	0.058	0.637	0.562
TL_Fr	931.837	937.837	945.120	938.077	941.051	0.057	0.686	0.569
Mo_Fr	933.382	939.382	948.601	939.622	942.596	0.060	0.665	0.602
Fr	934.565	938.565	945.873	938.684	940.708	0.080	0.577	0.801

## 8 Conclusion

In this article, we introduce a new extension of the Fréchet distribution, known as the new extended Fréchet (NE_Fr) distribution. Because of its attractive properties, it can construct asymmetrical probability density functions, making it ideal for modeling lifetime phenomena. Furthermore, it applies to many real-world data sets since the corresponding hrf has a decreasing or upside-down shape. The mathematical characteristics of the new distribution are derived by the computation of the quantile function, ordinary moments, incomplete moments, moment generating function, conditional moment, Bonferroni curve, and Lorenz curve. Several various measurements of entropy have been developed. The inferences from the NE_Fr distribution are explored using several well-established methods, such as maximum likelihood estimation, least squares and weighted least squares estimation, and Anderson-Darling estimation. The simulation demonstrated the computational efficacy of these techniques. The applicability of the newly suggested model is proven by examining three real-world data sets. A limitation of this paper is that it only uses complete samples to estimate the parameters of the NE_Fr distribution. As a result, several censoring techniques might be used to estimate the NE_Fr model's unknown parameters.

## CRediT authorship contribution statement

**Mohammed Elgarhy:** Writing – review & editing, Writing – original draft, Methodology, Formal analysis, Conceptualization. **Mohamed Kayid:** Writing – original draft, Resources, Investigation, Formal analysis, Conceptualization. **Ibrahim Elbatal:** Writing – review & editing, Validation, Supervision, Resources, Methodology, Formal analysis. **Mustapha Muhammad:** Writing – review & editing, Software, Formal analysis, Conceptualization.

## Declaration of Competing Interest

The authors declare that they have no conflict of interest.

## Data Availability

Any data that supports the findings of this study is included in the article.
